# Dynamic changes in microglial and macrophage characteristics during degeneration and regeneration of the zebrafish retina

**DOI:** 10.1186/s12974-018-1185-6

**Published:** 2018-05-28

**Authors:** Diana M. Mitchell, Anna G. Lovel, Deborah L. Stenkamp

**Affiliations:** Department of Biological Sciences, University of Idaho, 875 Perimeter Drive, MS 3051, Moscow, ID 83844-3051 USA

**Keywords:** Retina, Regeneration, Microglia, Macrophages, Zebrafish, Müller glia

## Abstract

**Background:**

In contrast to mammals, zebrafish have the capacity to regenerate retinal neurons following a variety of injuries. Two types of glial cells, Müller glia (MG) and microglia, are known to exist in the zebrafish retina. Recent work has shown that MG give rise to regenerated retinal neurons, but the role of resident microglia, and the innate immune system more generally, during retinal regeneration is not well defined. Specifically, characteristics of the immune system and microglia following substantial neuron death and a successful regenerative response have not been documented.

**Methods:**

The neurotoxin ouabain was used to induce a substantial retinal lesion of the inner retina in zebrafish. This lesion results in a regenerative response that largely restores retinal architecture, neuronal morphologies, and connectivities, as well as recovery of visual function. We analyzed cryosections from damaged eyes following immunofluorescence and H&E staining to characterize the initial immune response to the lesion. Whole retinas were analyzed by confocal microscopy to characterize microglia morphology and distribution. Statistical analysis was performed using a two-tailed Student’s *t* test comparing damaged to control samples.

**Results:**

We find evidence of early leukocyte infiltration to the retina in response to ouabain injection followed by a period of immune cell proliferation that likely includes both resident microglia and substantial numbers of proliferating, extra-retinally derived macrophages, leading to rapid accumulation upon retinal damage. Following immune cell proliferation, Müller glia re-enter the cell cycle. In retinas that have regenerated the layers lost to the initial injury (histologically regenerated), microglia retain morphological features of activation, suggesting ongoing functions that are likely essential to restoration of retinal function.

**Conclusions:**

Collectively, these results indicate that microglia and the immune system are dynamic during a successful regenerative response in the retina. This study provides an important framework to probe inflammation in the initiation of, and functional roles of microglia during retinal regeneration.

**Electronic supplementary material:**

The online version of this article (10.1186/s12974-018-1185-6) contains supplementary material, which is available to authorized users.

## Background

The mammalian retina is unable to regenerate damaged neurons due to acute trauma or degenerative disease. Rather than restoration of lost neurons, a gliotic response ensues, which is often associated with continual inflammation [1]. Inflammation is the body’s response to tissue injury and/or infection, and is initiated by the innate immune system. Inflammation includes activation of immune cells and the production of pro-inflammatory cytokines and molecular mediators. Particular cell types activated and cytokines/molecular mediators produced will vary depending on the initial insult or injury, the tissue location, and the immune response necessary to resolve the infection or injury. Within the mammalian retina, resident microglia rapidly sense changes in the microenvironment and can initiate and participate in both acute and chronic inflammatory responses [2]. Acute inflammation is crucial to clearing dead cells, debris, and pathogens, which must occur prior to subsequent initiation of tissue repair. However, when inflammation becomes chronic, it can be extremely damaging and contribute to tissue pathology. While chronic inflammation and immune activation in mammalian neurodegenerative disease is well appreciated, the role of the immune system in contexts of successful retinal regeneration has not been well explored.

In contrast to mammals, teleost fish have a remarkable capacity to regenerate damaged retinas following a variety of lesions that destroy neurons [3–5]. The source of regenerated retinal neurons in zebrafish are the Müller glia (reviewed in [6–8]), retinal glial cells also present in mammals and other vertebrates. Upon injury, Müller glia re-enter the cell cycle [9–13] and undergo an asymmetric division [14], ultimately generating multipotent progenitors that replace lost retinal neurons [11, 14–16]. Intriguingly, Müller glia in mammals and other vertebrates appear to have properties suggesting regenerative potential, including the upregulation of progenitor markers and cell cycle re-entry (reviewed in [1, 4, 17–19], but this instead results in gliosis due to the inability to differentiate into neurons (extensively reviewed in [1, 17]). Further, immune-Müller glial crosstalk may be important in shaping the Müller glia response to retinal injury [20–22].

In wound healing outside of the central nervous system, the role of the immune system is well appreciated. In general, a period of degeneration is followed by regeneration in which the lost cell types are replaced and tissue structure is restored. Tissue resident macrophages are crucial to coordinating the clearance of damaged tissue as well as instructing differentiation of new cell types to replace damaged tissue [23]. Resident macrophages as well as recruited phagocytes, such as neutrophils, monocyte-derived macrophages, and macrophages that migrate from other tissues participate in the phagocytosis and clearance of degenerative debris before a regenerative response begins [23]. In zebrafish, recruited macrophages appear to be essential to regeneration of peripheral nervous system tissue [24, 25]. The role of the innate immune system and resident macrophages, microglia, in regeneration of the central nervous system in organisms which have such regenerative capacity, such as teleost fish, is not well defined. Interestingly, a recent publication using larval zebrafish supports a role for inflammation and resident microglia in regeneration of rod photoreceptors in the retina [26]. Inflammation has also been shown to be important in regeneration of the adult zebrafish brain [27]. Yet, it is not clear if these findings translate to retinal regeneration in an adult animal.

Under normal conditions, retinal microglia are highly ramified and integrated among the highly organized retinal tissue. Microglial responses to retinal insult and degeneration have been studied mainly in rodent model systems [28]. Generally, when responding to neurological insult, microglia transform morphology from ramified to an ameboid shape and migrate to sites of cell death [29, 30]. However, microglial responses to retinal injury in zebrafish have not been well documented, and it remains unclear under which conditions of retinal injury extra-retinal immune cells may participate in the initial response to neuronal degeneration. Studies to date in zebrafish have focused on how inflammation, specific cytokines, and microglia/macrophages may affect the proliferation of Müller glial cells, which give rise to regenerated retinal neurons [22, 26, 31]. There has been a surge of interest in the role of microglia in development and maintenance of the central nervous system, as well as their involvement in, and possibly sources of, neurodegenerative disease [2, 32]. Yet, little is known about the role of microglia in initiating and/or shaping successful regeneration of retinal neurons, and if these functions are shared with or unique from those in development.

In the present study, we use a tissue-disrupting lesion, intraocular injection of the neurotoxin ouabain, to destroy inner retinal neurons while sparing glia and photoreceptors [9, 10, 14, 33]. This type of lesion is known to result in a regenerative response that largely restores retinal architecture, neuronal morphologies and connectivities, and behavioral and electrophysiological measures of retinal function [10, 33]. We characterize the initial response to this cytotoxic damage to determine how the innate immune system responds to retinal degeneration that is subsequently followed by a successful regenerative response. We then investigate microglial characteristics, including distribution, morphology, selective markers, and histological features, in regenerated retinas. We find that in damaged zebrafish retinas, both resident and infiltrating immune cells contribute to mount a robust response to neuronal cell death that is immediately followed by Müller glia proliferation. Upon histological regeneration of retinal layers, resident microglia localize to regions of regenerated neurons and maintain morphologies indicative of ongoing functional activation. Our findings indicate that retinal regeneration in adult zebrafish provides an excellent system to discover functional roles for the immune system and microglia that may be crucial to supporting successful regenerative responses.

## Methods

### Animals

Procedures involving zebrafish were performed in compliance with protocols approved by the University of Idaho Animal Care and Use Committee (IACUC). Zebrafish (*Danio rerio*) were maintained on a 14:10 light/dark cycle in 28.5 °C recirculating, monitored system water, housed, and propagated according to [34]. Zebrafish transgenic lines used in this study include *mpeg1:GFP* (*gl22 Tg*, GFP expressed in microglia/macrophages [35], available from Zebrafish International Resource Center, ZIRC); *mpeg1:mCherry* (*gl23 Tg*, mCherry expressed in microglia/macrophages [35], available from ZIRC). The *gl22 Tg* and *gl23 Tg* transgenic lines co-label cells in retinal tissue (Additional file 1: Figure S1). The wild-type strain used, referred to as SciH, was originally obtained from Scientific Hatcheries (now Aquatica Tropicals).

### Retinal lesion

Chemical lesioning of zebrafish retinas (age 6–14 months, both sexes) was performed by intravitreal injection of ouabain in order to destroy inner retinal neurons and spare photoreceptors and Müller glia [9, 10, 33]. A working stock of 40 μM ouabain (ouabain octahydrate, Sigma-Aldrich) was prepared in 0.65% sterile saline (NaCl) solution. Fish were anesthetized by immersion in tricaine solution, and an incision was made across the cornea using a sapphire blade. A Hamilton syringe was inserted behind the lens and 0.4–0.6 μL of 40 μM ouabain solution was injected into the vitreal chamber. Volume injected was based on diameter of the eye (measured with calipers) and upon calculations based on geometry and volumes of the eye and lens, resulting in an estimated intraocular concentration of 2 μM [9, 36]. Lesions were unilateral and only the right eye was injected. For control samples, right eyes of a separate group of fish were injected with 0.65% sterile saline (NaCl) solution. The same sterile saline solution was used for saline injections and for preparation of ouabain solution for injection. During the procedure, fish were continuously flushed with tricaine solution. Immediately following the procedure, fish were returned to tanks with clean system water. For all experiments, 3 control (saline injected) and 3–4 ouabain injected fish were used for subsequent analysis.

### Tissue collection and processing

At selected time points post-injection, tissue samples were collected for analysis. For whole retina collection, fish were dark adapted for approximately 12 h, anesthetized in tricaine solution, and then eyes were enucleated using fine forceps, and placed into phosphate-buffered (pH = 7.4) saline (PBS) for dissection. The cornea and lens were removed and retinas were peeled from the whole eyecup. The retinal pigmented epithelium (RPE) detached from the retina due to the dark adaption, and any remaining RPE was brushed away using a soft paintbrush. Retinas were then rinsed several times in fresh PBS using a plastic transfer pipette, transferred into fixative consisting of 4% paraformaldehyde in PBS for approximately 1 h at room temperature with constant gentle agitation, then washed several times with PBS containing 0.01% TritonX-100 (PBST).

To prepare retinal cryosections, whole eyes were enucleated using fine forceps, transferred to PBS, and the lens was removed. Eyes were then fixed in phosphate-buffered, 4% paraformaldehyde containing 5% sucrose for 1 h at room temperature, washed in phosphate-buffered (pH = 7.4) 5% sucrose, and then washed in a graded series ending in 20% sucrose. The following day, tissues were embedded in blocks of a 1:2 solution of OCT embedding medium (Sakura Finetek) and phosphate-buffered, 20% sucrose, and frozen in isobutane supercooled with liquid N_2_. After freezing solid, tissues were sectioned at 5 μm thickness using a Leica CM3050 cryostat. After overnight desiccation, tissue sections on glass slides were stored at − 20 °C until use.

To evaluate co-label of *gl22 Tg* and *gl23 Tg*, whole embryos were fixed at 3 days post-fertilization and washed with PBST as described for whole retina collection above. Whole eyes were removed and mounted in  VECTASHIELD (Vector Labs), covered with a coverslip, then imaged as described below.

### Immunofluorescence

For staining of whole retinas, fixed tissue was blocked in 20% goat serum for 1 h at room temperature, incubated with primary antibody for 1–3 days at 4 °C, washed three times in PBST, then incubated with conjugated secondary antibody and DAPI (4′,6-diamidino-2-phenylindole, 4.25 μM) for 1–3 days at 4 °C, washed twice in PBST with a final wash in PBS. All antibody incubations and washes were performed with constant agitation. Whole retinas were then flat mounted by cutting four slits around the perimeter of the retinal cup and were mounted flat onto a microscope slide in VECTASHIELD (Vector Labs), covered with a coverslip, and sealed with clear nail polish.

To stain cryosections, tissue sections (5 μm thickness) were blocked in 20% goat serum for 30 min at room temperature, incubated in primary antibody overnight, washed in PBST for at least 30 min, incubated in secondary antibody for 1 h, and washed in PBST for at least 30 min. Slides were then mounted in Vectashield + DAPI (Vector Labs), covered with a coverslip, and sealed with clear nail polish. Primary antibodies and dilutions used rabbit polyclonal anti-zebrafish L-plastin [37, 38] (1:10,000, a kind gift of Dr. Michael Redd), rabbit anti-zebrafish mpx (1:200, Genetex), mouse ZPR1 antibody (labeling cone-arrestin 3a [39], 1:200, ZIRC), mouse anti-PCNA (PC10, 1:200, Santa Cruz Biotech), rat anti-PCNA (16D10, 1:200, Chromotek), rabbit anti-phosphorylated histone 3 (PH3) (1:500, Cell Signaling), rabbit polyclonal anti-PKCα (Santa Cruz Biotech), mouse ZRF1 antibody (labeling GFAP, 1:200, ZIRC), and mouse anti-Glutamine Synthetase (1:1000, BD Transduction Laboratories). Secondary antibodies conjugated to Cy3, FITC, or Alexa-Fluor647 (Jackson ImmunoResearch) were used at 1:200 dilution.

### H&E staining

Slides containing cryosections were air dried at room temperature for 30 min, immersed in 0.1% Hematoxylin (MHS-16, Sigma-Aldrich) 5 min, then washed in running tap water for 5 min. Slides were then immersed in 0.5% eosin (Sigma-Aldrich) for 1 min, rinsed in deionized water until rinse was clear, then immersed in a series of ethanol solutions (50, 75, 95, and 100%) for 30 s-1 min each, dipped five times in Xylenes (Fisher Scientific) and immediately mounted with Permount (Fisher Scientific) and sealed with a coverslip.

### TUNEL staining

TUNEL (Terminal deoxynucleotidyl transferase dUTP nick end labeling) staining of 5 μm cryosections was performed following the manufacturer’s instructions (Roche). Briefly, cryosections were washed for 20 min in × 1 PBS at room temperature then placed in ice-cold permeabilization solution for 2 min. Slides were washed twice in × 1 PBS, 10 min per wash, incubated in TUNEL reaction mixture at 37 °C in the dark for 3 h, then rinsed three times in PBS. Immediately following, cryosections were blocked in 20% goat serum for 30 min at room temperature and subsequent immunofluorescence was performed as described above.

### Microscopy and image acquisition

Imaging of whole flat mounted retinas (z series) or fluorescently stained retinal sections (single plane) was performed with a Nikon Andor spinning disk confocal microscope equipped with a Zyla sCMOS camera running Nikon Elements software. Imaging was performed using × 20 (dry) or × 40 (oil immersion) objectives. For stitched images of entire retinal cryosections, images were acquired at × 20 magnification using the large stitched images feature in Nikon Elements software and stitched based on DAPI staining. Z stacks of flattened retinas were generally obtained at 1 μm intervals. Imaging of H&E stained cryosections was performed using a Leica DM2500 compound microscope with Leica DFC700T color camera using × 20, × 40 (dry), or × 100 (Oil) objectives. Image processing and analysis was performed using FIJI (ImageJ).

### Quantification of immune cells in retinal cryosections (24–72 h post-injection)

Using FIJI (ImageJ), a region of interest was drawn in each image obtained from cryosections corresponding to the region of the retinal lesion in the inner retina: from the basal border of the outer nuclear layer to the basal border of the ganglion cell layer. For 48 and 72 h post-injury (hpi) time points, the basal layer of this border was defined based on the limit of dense DAPI staining. DAPI+ nuclei surrounded by L-plastin+ cell bodies were counted within the defined region of interest and normalized per 1000 square microns. Note that the analyzed region corresponds to the lesioned inner retina where the *x* direction is parallel to retinal layers and the *y* direction is perpendicular to retinal layers. Since the inner nuclear layer is damaged due to the ouabain-induced lesion and becomes thinner, the number of L-plastin+ cells is also reported per 100 μm of curvi-linear distance of retina using a line that follows parallel to the basal border of the ONL. Three to four non-adjacent cryosections were analyzed per fish.

### Quantification of immune cells in whole, flat mounted retinas (histologically regenerated retinas)

Using FIJI (Image J), individual microglia (DAPI+ nuclei surrounded by L-plastin+ signal) in flattened whole retinas were counted in individual image stacks containing all *z* planes obtained at × 40 magnification, from the vitreal face of the ganglion cell layer to the apical face of the outer nuclear layer. Counts were normalized per 1000 square microns of flattened retina where the *x* and *y* direction are parallel to retinal layers. Counts were normalized to area rather than volume because the regenerated retinas are thinner than controls [33]. Six to eight images covering approximately 190,000 μm² per image were analyzed from each whole retina.

### Analysis of microglia morphology

We analyzed morphology of microglia in the ganglion cell layer of histologically regenerated and control retinas. Confocal *z* stacks obtained from whole, flat mounted retinas using a × 40 Oil objective corresponding to the region of the ganglion cell layer were flattened using FIJI (ImageJ). We used the marker L-plastin, which is a leukocyte-specific form of the actin bundling protein plastin expressed by zebrafish macrophages [37], to analyze microglia in control and histologically regenerated retinas because (1) all retinal L-plastin+ cells co-label with GFP expression in *mpeg1*:GFP transgenic fish at these time points and (2) L-plastin staining labels the entire cell body and cellular processes continuously, whereas the *mpeg1* promoter-driven transgenic reporter labels portions of the cell (Fig. 9A, A’, B, B’). Outlines of individual L-plastin+ cells were manually traced and measurements of morphological features were analyzed using the “measure” tool in FIJI. Approximately 35 microglia, taken from 3 to 4 retinas per condition, were analyzed for each condition and histograms of the distributions were created. Descriptions of the parameters used to analyze microglia morphology are as follows. Area: area of an individual microglia in square microns; circularity: circularity of an individual microglial cell body. A value of 1.0 indicates a perfect circle, while values closer to 0 indicate elongated shapes; perimeter: the total length of a line tracing the outside boundary of an individual microglial cell in microns; Feret diameter, also known as maximum caliper, the longest distance between any two points along the perimeter of an individually traced microglial cell.

### Statistical analysis

A two-tailed Student’s *t* test was used to obtain *p* values comparing saline injected to ouabain injected samples. *P* values less than 0.05 are reported in the figures.

## Results

### Immune cell response to a tissue-disrupting retinal lesion

**Fig. 1:**
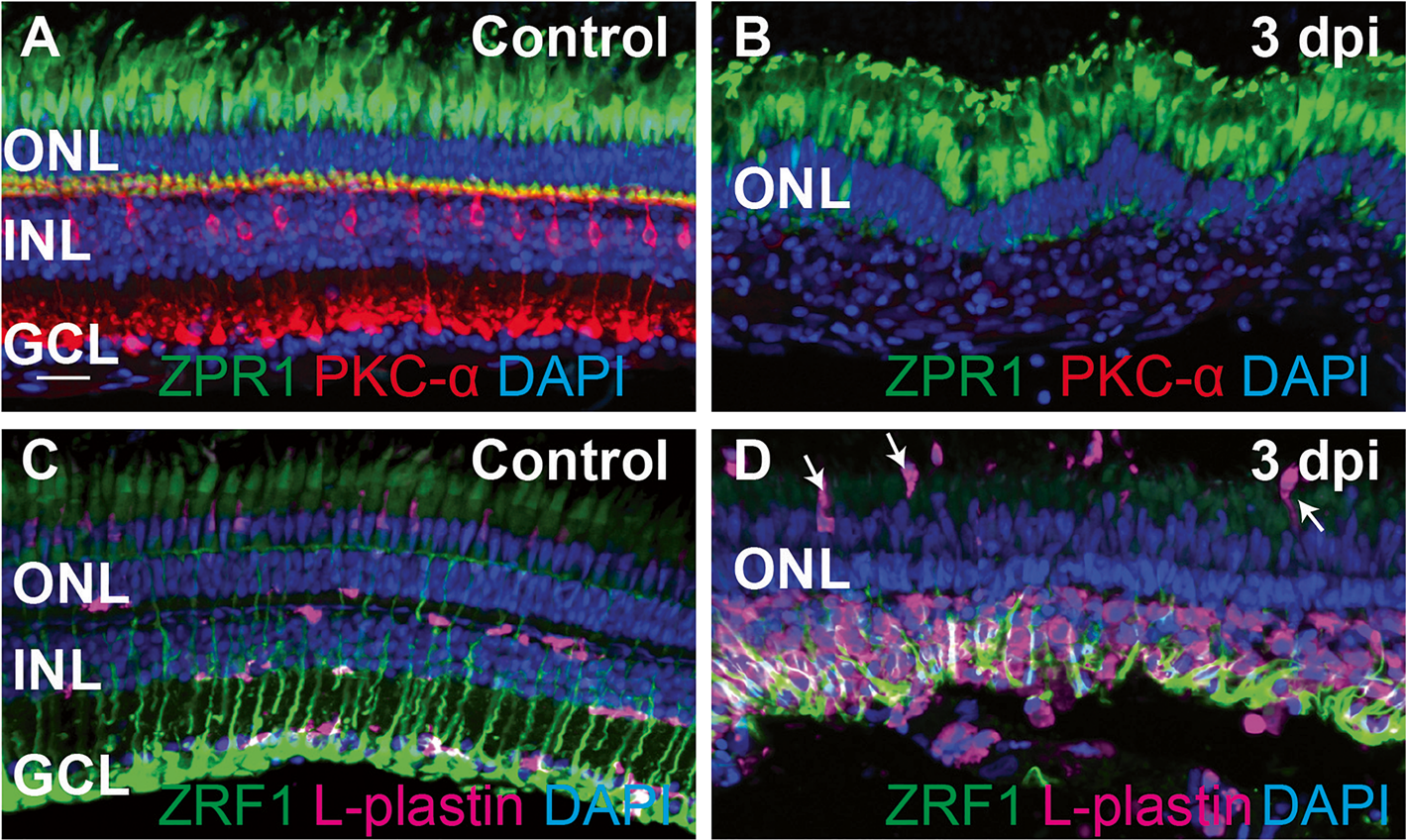
Ouabain-induced retinal degeneration results in a robust accumulation of responding immune cells. Images show cryosections from undamaged (control, **A**, **C**) and ouabain-damaged retinas at 3 days post-injection (3 dpi, **B**, **D**). **A** ZPR1 (green), PKC-α (Red), and DAPI (blue) were used to label cones of the outer retina, bipolar neurons of the inner retina, and all nuclei, respectively, in undamaged retinas. **B** Images of damaged retinas sampled at 3 dpi; inner retinal neurons have been destroyed (note the absence of PKC-α staining and absence of DAPI+ layer corresponding to the ganglion cell layer; GCL), but photoreceptors are spared (ZPR1, green). **C** Ramified microglia, labeled by L-plastin (magenta), are present in undamaged retinas, along with radially patterned Müller glia (ZRF1, green). **D** Müller glia (green) are spared from the ouabain-induced lesion. A large number of immune cells (magenta) responding to the lesion are present in the damaged retina, primarily localized to the region of neuronal cell death (inner retina), although some appear to be recruited from regions apical to the retina (arrows). DAPI+ nuclei in the inner retina visible in **B** can be identified as immune cells and Müller glia. Scale bar in **A**, applies to all images, = 20 μm. ONL = outer nuclear layer, INL = inner nuclear layer, GCL = ganglion cell layer

**Fig. 2:**
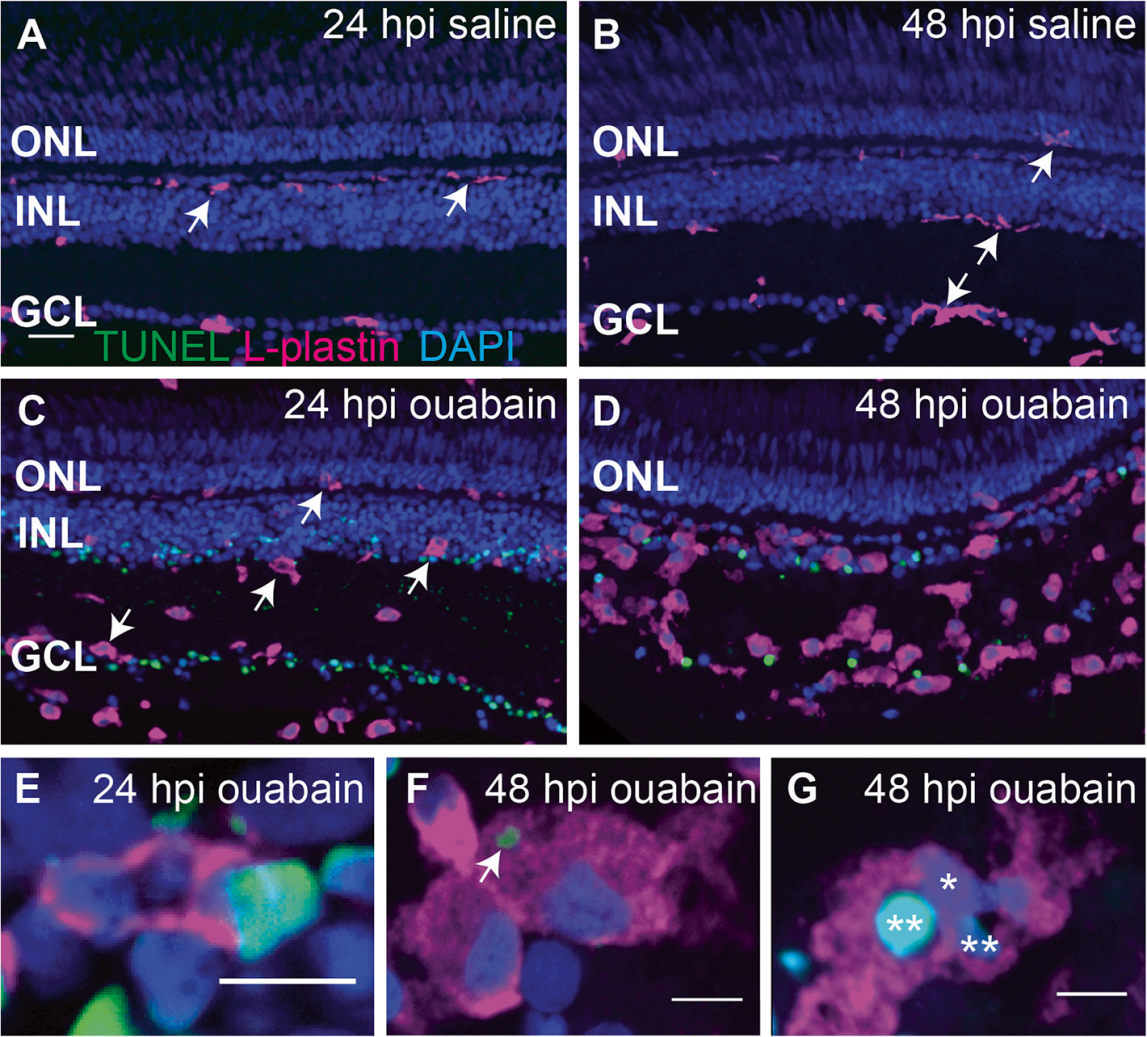
Progression of ouabain-induced retinal cell death and accumulation of immune cells in damaged retinas. Cryosections (5 μm thick) from retinas at 24 and 48 h post-intravitreal injection (24 and 48 hpi) of saline (**A** and **B**) or 2 μM ouabain (**C** and **D**) were stained using the TUNEL cell death detection process (green), the immune cell marker L-plastin (magenta), and DAPI (blue). **A**–**B** Saline injection did not induce cell death (absence of TUNEL staining), and immune cells remain ramified (arrows). **C**–**D** TUNEL+ nuclei and debris (green) are present in the ganglion cell layer (GCL) and inner nuclear layer (INL) following intravitreal injection of 2 μM ouabain, and immune cells assume ameboid morphologies (arrows). As the lesion progresses over time, immune cells accumulate in regions of cell death, and the clearance of TUNEL+ nuclei and debris corresponds to this immune cell accumulation. Few immune cells contained TUNEL+, DAPI+ nuclei (quantification in the “Results” section), suggesting that immune cell death was not significantly induced by ouabain. **E**–**G** High magnification images of immune cells revealed immune cells phagocytosing TUNEL+ nuclei (**E**), immune cells with cytoplasmic TUNEL+ material (**F**, arrow), and immune cells with multiple nuclei, some of which are TUNEL+ (**G**, asterisks), suggesting that the accumulating immune cells are highly phagocytic and important for clearing dead cells and debris from the lesioned retina. Scale bar in **A** (applies to **A**–**D**) = 20 μm. Scale bars in **E**, **F**, and **G** = 5 μm. ONL = outer nuclear layer, INL = inner nuclear layer, GCL = ganglion cell layer

To investigate the immune response to substantial neuronal damage in the retina, we used intravitreal injection of the neurotoxin ouabain to generate this damage. Injection of ouabain to a final concentration of 2 μM causes death of inner retinal neurons, as demonstrated by the loss of the PKCα+ bipolar neuron population at 3 days post-injection (dpi) (Fig. 1) and appearance of TUNEL+ nuclei within the inner retina at 24 and 48 h post-injection (hpi) (Fig. 2). Photoreceptors and glia are spared [9, 10, 14, 33], as demonstrated by the continued presence of zpr1+ cones and zrf1+ Müller glia at 3 dpi (Fig. 1). We visualized microglia/immune cells in control and ouabain-lesioned retinas using an antibody to zebrafish L-plastin, which is used to mark leukocytes, including macrophages, in zebrafish [37, 38]. Similar to reports in embryonic and larval zebrafish [40], microglia in intact, undamaged adult zebrafish retina reside in the inner nuclear layer, ganglion cell layer, and nerve fiber layer (Fig. 1C). In addition, microglia flank the apical side of the inner plexiform layer and the apical and basal sides of the outer plexiform layers (Fig. 1C). This is different than reports from mammals, which show that microglia localize within plexiform layers [2]. After intravitreal injection of ouabain, we found a dramatic accumulation of immune cells in the region of the lesion by 3 dpi (72 hpi) (Fig. 1D). At 3 dpi, nearly all inner retinal neurons have been destroyed, Müller glia are reactive, and some Müller glia have re-entered the cell cycle ([9, 10, 14] and Fig. 1D). Many DAPI+ nuclei are present in regions of neuronal death and degeneration of the inner retina (Fig. 1B, D), and essentially all of these DAPI+ nuclei at 72 hpi correspond to immune cells and reactive Müller glia (Fig. 1D).

To monitor the accumulation of immune cells over the course of ouabain-induced cell death and to determine how ouabain and the intravitreal injection process affect immune cells in the retina, we examined L-plastin and TUNEL labeled cryosections from ouabain injected retinas at 24 and 48 hpi, compared to saline injected controls (Fig. 2). In ouabain lesioned retinas, TUNEL+ nuclei and TUNEL+ debris within the inner retina appear by 24 hpi, concomitant with immune cell accumulation and activation as indicated by morphological changes of L-plastin+ cells from ramified to ameboid cell shape (Fig. 2A, C). Immune cells in retinas from saline injected controls remained ramified, and in their typical locations, and TUNEL signal was not detected (Fig. 2A, B). By 48 hpi, immune cells with ameboid morphology continue to accumulate in locations corresponding to ouabain-induced neuronal death and degeneration (INL, GCL, Fig. 2D). This accumulation correlates with a decrease in TUNEL+ nuclei and debris from 24 to 48 hpi (Fig. 2C, D), indicating that immune cells have cleared debris resulting from neuronal death. Consistent with this, we observed immune cells engulfing TUNEL+ nuclei (Fig. 2E) as well as TUNEL+ puncta within L-plastin+ immune cell bodies (Fig. 2F). However, only a small fraction of L-plastin+ cells contained TUNEL+, DAPI+, co-labeled nuclei (8.4 ± 5.7% at 24 hpi and 1.9 ± 1.9% at 48 hpi), indicating that ouabain only induced minor levels of immune cell death. Further, some of the DAPI and TUNEL co-labeling within immune cells could potentially be explained as phagocytosed TUNEL+ nuclei from dead or dying neurons. Consistent with this, in some cryosections, we observed multiple nuclei, including TUNEL-nuclei, surrounded by L-plastin+ cytoplasm (Fig. 2G).

### Origins and distributions of immune cells in damaged retina

**Fig. 3:**
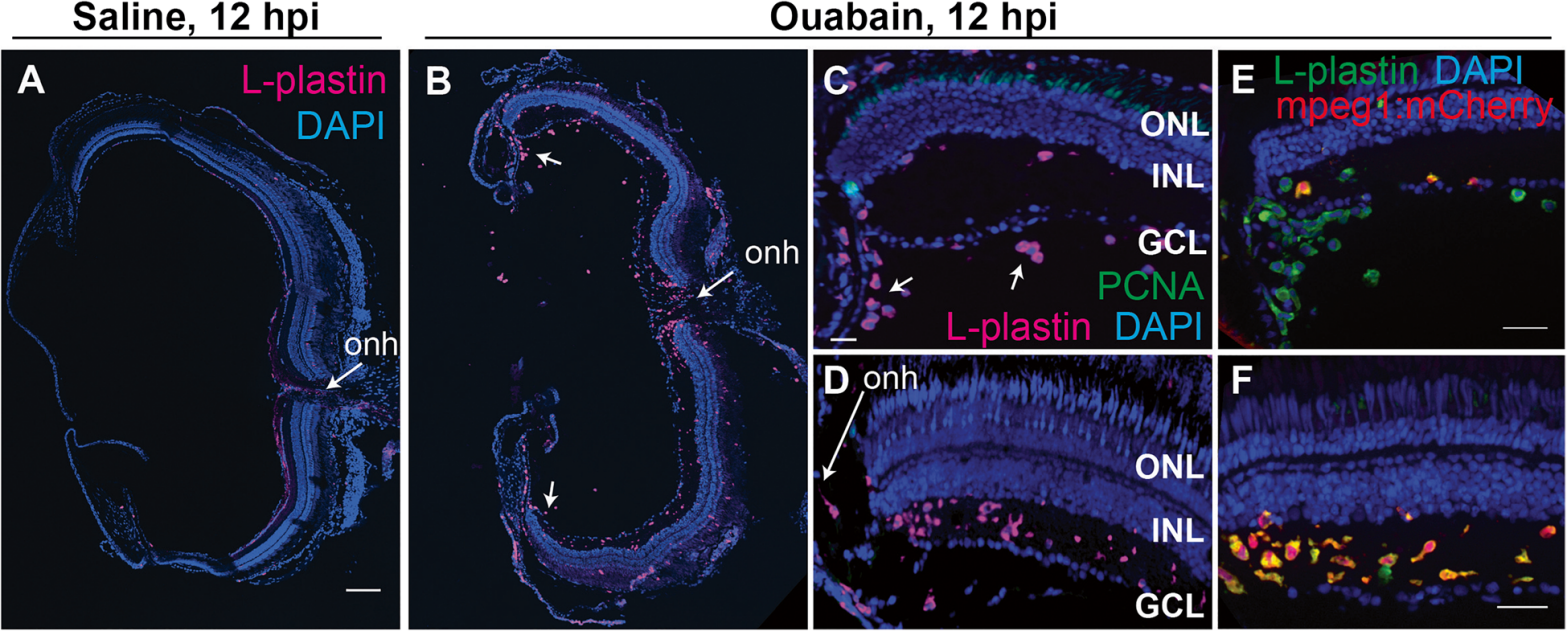
Evidence of early infiltration of immune cells to the retina at 12 h post-ouabain injection. Images of retinal cryosections at 12 h post-saline (**A**), or ouabain (**B**–**F**) injection (12 hpi). **A**, **B** Show stitched images of entire cryosections stained for L-plastin (magenta) and DAPI (blue) obtained at × 20 magnification. Images in **C** and **E** show peripheral regions of retina. **D, f** Show central regions of retina adjacent to the optic nerve head (onh). Following ouabain injection, ameboid immune cells are seen in a gradient originating from the region of the optic nerve head (**B**, **D**, **F**), as well as vitreal to the ganglion cell layer in peripheral regions (**C** (arrows) and **E**). Cryosections were also stained for PCNA (green, **C** and **D**). PCNA expression was only rarely detected at this time point. **E** In peripheral retina, few L-plastin+ cells (green) co-label with mpeg1:mCherry transgene expression (red, co-labeled cells yellow). **F** In central retina, most L-plastin+ cells (green) co-label with mpeg1:mCherry transgene expression (red, co-labeled cells yellow). **A**–**D** were obtained at × 20 magnification, **E** and **F** were obtained at × 40 magnification. Scale bar in (**A**) = 100 μm (applies to **A** and **B**); scale bar in **C** (applies to **C** and **D**) = 20 μm; scale bars in **E** and **F** = 20 μm

To better understand the origins of the accumulation of immune cells in ouabain-lesioned retinas, we imaged retinal cryosections at 12 hpi using the marker L-plastin and the S-phase marker PCNA (Fig. 3). Saline injection did not result in immune cell accumulation or PCNA expression (Fig. 3A and Additional file 1: Figure S2). At 12 hpi following ouabain injection, ameboid immune cells can be seen in central retina in a gradient originating from the region of the optic nerve head (Fig. 3B, D, optic nerve head indicated) and vitreal to the ganglion cell layer in peripheral retina (arrows, Fig. 3B, C). We did not detect substantial PCNA signal in immune cells at this time point (Fig. 3C, D), indicating that the resident microglia did not immediately enter the cell cycle. Cells of the macrophage lineage (including microglia) in zebrafish have been identified using the transgenic expression of *mpeg1-*driven transgenes [35], while neutrophils are identified by expression of *mpx* (also called *mpo*) [41]. Nearly all of the L-plastin+ cells co-labeled with *mpeg1:mCherry*, indicating that the majority of these leukocytes are macrophages (Fig. 3E, F). Few mpx + neutrophils were detected within the peripheral leukocyte population vitreal to the ganglion cell layer at this time point; however, mpx + neutrophils were not detected in other retinal regions, including regions surrounding the optic nerve head (Additional file 1: Figure S3). The presence of few neutrophils supports that leukocytes in the blood have access to retinal tissue following ouabain injection. Previous reports indicate that apoptotic cells labeled by TUNEL are present as early as 3 hpi ouabain injection, although the number of apoptotic cells peaks at approximately 24 hpi [9]. The presence of ameboid immune cells apparently infiltrating the retina, and absence of PCNA expression, suggests an early infiltration of immune cells following ouabain injection, prior to the peak of retinal cell death.

**Fig. 4:**
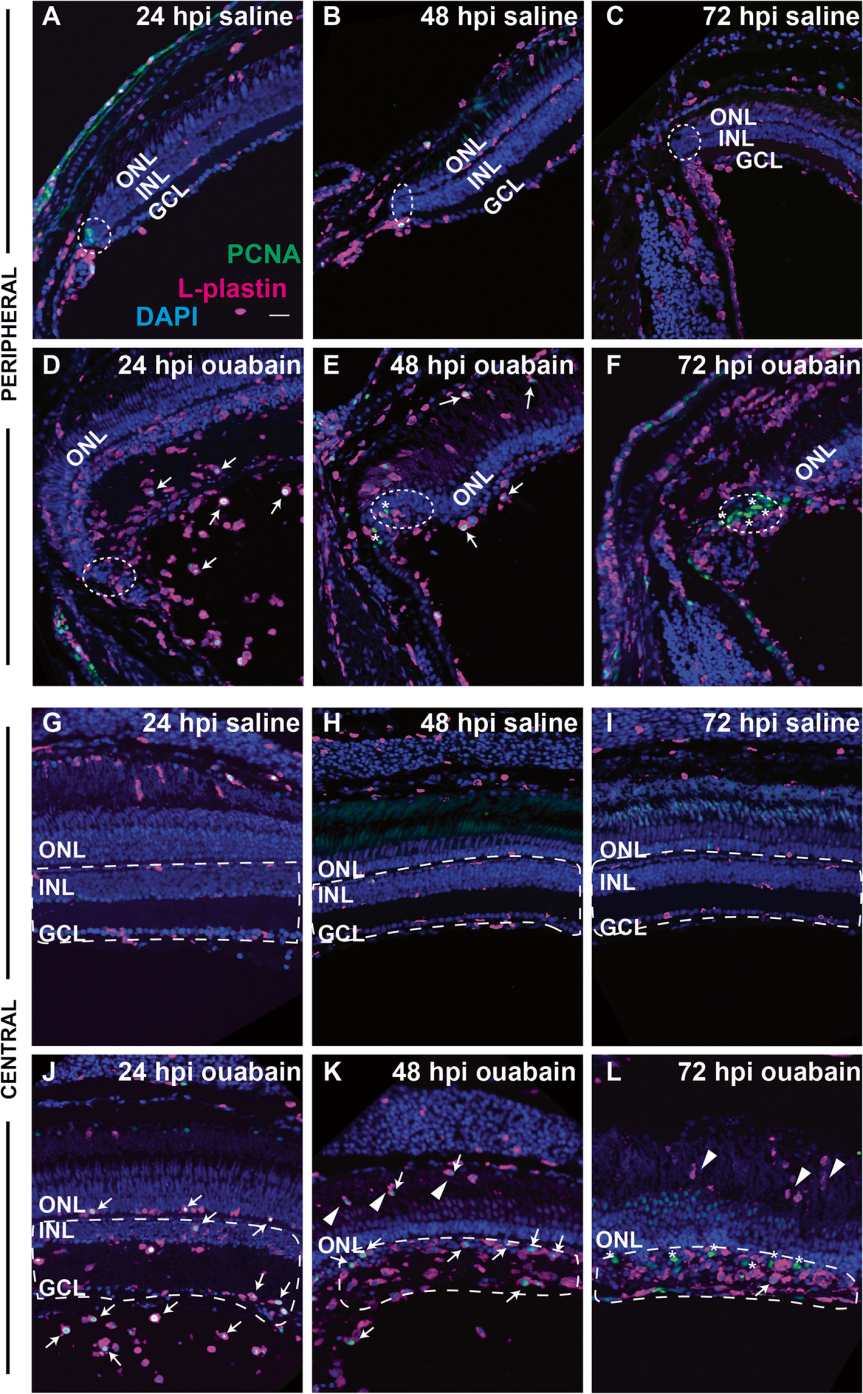
Distribution and proliferation markers in immune cells during the response to retinal damage. Images show staining of L-plastin (magenta), PCNA (green), and DAPI (blue) in cryosections of retinas injected with saline (**A**–**C** and **G**–**I**) or ouabain (**E**–**F** and **J**–**L**) at 24, 48, and 72 hpi. Images are from regions of peripheral retina (rows 1 and 2, **A**–**F**) or central retina (rows 3 and 4, **G**–**L**). PCNA+, L-plastin+ cells are indicated with white arrows, while PCNA+ non-immune cells are indicated with asterisks. In images of peripheral retina, regions corresponding to the ciliary marginal zone (CMZ) are within dotted ellipses. **A**–**C** and **G**–**I** Saline injection does not result in significant changes in L-plastin+ cell accumulation, location, or morphology and does not result in significant PCNA+ signal in L-plastin+ cells (**A**–**C** and **G**–**I**). **D**–**F** and **J**–**L** In ouabain-lesioned retinas, significant numbers of ameboid-shaped L-plastin+ cells are located in the region vitreal to the ganglion cell layer at 24 hpi and many are PCNA+ (**D**, **J**, arrows). At 48 hpi ouabain, L-plastin+ cells remain ameboid in shape, appear to accumulate at the region of the ouabain-induced lesion, and many are PCNA+ (**E** and **K**, arrows). By 72 hpi, PCNA+ signal is mainly localized to non-immune cells (asterisks*), although ameboid-shaped L-plastin+ cells remain in regions corresponding to the lesion (**F** and **L**). **K**, **L** Arrowheads indicate immune cells invading from regions posterior to the retina. **G**–**L** Regions denoted with dotted white lines indicate regions (corresponding to the lesion) used for quantification of L-plastin+ cells shown in Fig. 4. Scale bar in **A** (applies to all images) = 20 μm. ONL = outer nuclear layer, INL = inner nuclear layer, GCL = ganglion cell layer

**Fig. 5:**
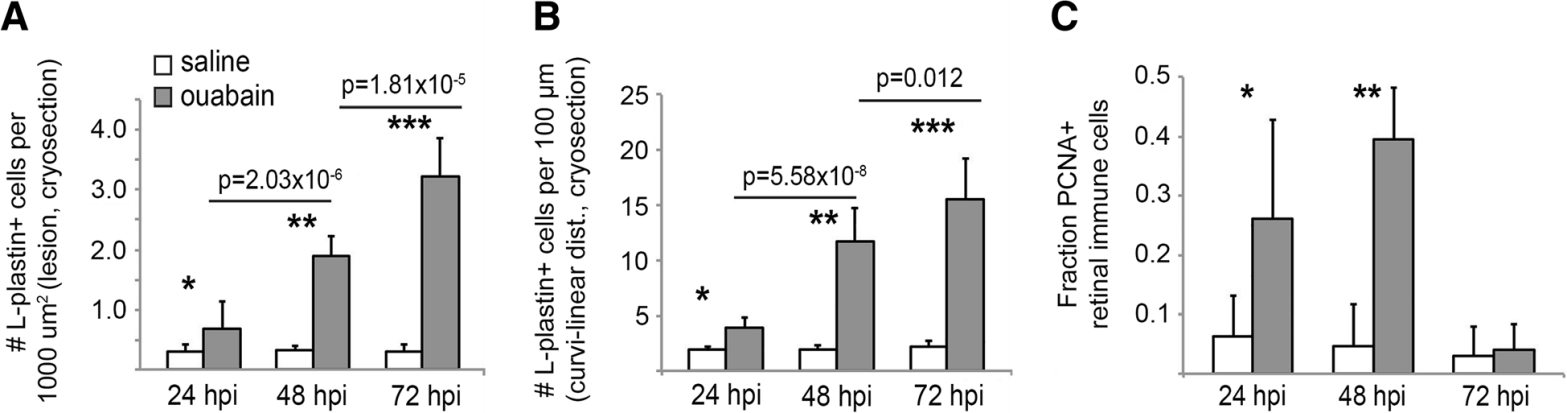
Quantification of immune cells in cryosections of damaged retina. **A** Quantification of L-plastin+ immune cells per 1000 μm² of inner retina (5-μm-thick sections) from saline or ouabain injected eyes at 24, 48, and 72 hpi, representative images in Fig. 3G–L). For details, see the “*Quantification of Immune Cells in Retinal Cryosections (24–72 h post-injection*)” section. Regions depicted by dotted white lines in Fig. 3G–L are representative regions used for quantification. DAPI+ nuclei surrounded by L-plastin+ signal were counted within the region of interest and normalized per 1000 μm². *p* values comparing densities between 24 and 48 hpi and 48–72 hpi ouabain injection are shown on the graph; asterisks indicate the following *p* values comparing ouabain to saline injected at indicated time points: *p = 0.012, **p = 7.84 × 10^− 12^, ***p = 1.21 × 10^− 12^. **B** Since thickness of the INL changes due to ouabain-induced lesion, the number of L-plastin+ cells is also reported per 100 μm of curvi-linear distance of retina using a line that follows parallel to the basal border of the ONL. *p* values comparing densities between 24 and 48 hpi and 48–72 hpi ouabain injection are shown on the graph; asterisks indicate the following *p* values comparing ouabain injected to saline injected at indicated time points: *p = 3.84 × 10^− 6^, **p = 8.48 × 10^− 9^, ***p = 5.5 × 10^− 10^. **C** PCNA+, DAPI+ nuclei surrounded by L-plastin+ signal quantified in retinal cryosections from saline or ouabain injected eyes at 24, 48, and 72 h hpi. Graph shows the fraction of L-plastin+ cell bodies with nuclear co-label of DAPI and PCNA. Only images from central and central-peripheral retina were quantified, and only L-plastin+ cell bodies within retinal tissue boundaries were counted. Error bars indicate standard deviation. *p = 0.0005, **p = 1.01 × 10^− 12^, comparing ouabain injected to saline injected at indicated time points

We next imaged cryosections at 24, 48, and 72 hpi using the markers L-plastin and PCNA (Fig. 4), and quantified numbers of cells stained by these markers (Fig. 5). Injection of saline did not result in accumulation of immune cells or significant expression of PCNA within the retina through 72 hpi (Figs. 4A–C, G–I, and 5A–C). By 24 h post-ouabain injection, immune cells appear abundant in the region vitreal to the degenerating ganglion cell layer in both peripheral and central retina (Fig. 4D), suggesting that extra-retinal immune cells continue to invade the retina in response to neuronal degeneration. Immune cells with ameboid shape begin to accumulate in regions of inner retina corresponding to the ouabain-induced lesion at 24 hpi and continue to accumulate through 72 hpi (Figs. 1D, 4D–F and J–L, 5A, B). Further, evidence of immune cell infiltration to the damaged retina remains at 48–72 hpi, but this later migration appears to originate from regions and structures apical to the neural retina, which could include the RPE or sclera and associated blood vessels (Fig. 4K, L). We consider this to be the case, because immune cells that appear directionally oriented to migrate into the retina (i.e., radially oriented) are more frequently present in the outer retina and RPE (Figs. 1D, 4K, L).

A significant fraction of immune cells responding to the ouabain-induced lesion at 24 and 48 hpi stain positive for the S-phase marker PCNA (Fig. 4, arrows, and Fig. 5C). PCNA+ immune cells are located in the vitreal space at 24 hpi ouabain injection (Fig. 4D), apical to the retina at 48 hpi (Fig. 4E, K), and within retinal tissues at both 24 and 48 hpi (Fig. 4D, E, J, K), suggesting that by 24 hpi, both resident and infiltrating immune cells have entered the cell cycle in response to the lesion. Importantly, the number of immune cells located within retinal tissues more than doubles between 24 and 48 hpi (Fig. 5A, B) while fewer than 50% of these cells are PCNA+ at 24 hpi (Fig. 5C). This rate of PCNA expression is not sufficient to account for all of the accumulated immune cells, supporting the interpretation that at least a portion of them infiltrated the retina. Numbers of PCNA+ immune cells decrease at 72 hpi and instead PCNA positive signal is present in non-immune cells (Fig. 4F, L asterisks, Fig. 5).

Using the M-phase specific marker PH3, we did not identify significant numbers of PH3+ cells at any of these time points (Additional file 1: Figure S4 and not shown). The absence of PH3+ cells could possibly be due to rapid mitosis, making detection of mitotic cells in fixed retinal tissue difficult. An alternative explanation, that immune cells die after S phase and prior to M phase, is not supported by the observations of very few L-plastin+, TUNEL+ cells (discussed above) and of the continued accumulation of immune cells through 72 hpi (Fig. 5A, B). These cell cycle markers indicate that infiltrating immune cells prepare for division upon their arrival to damaged retinal tissue, and resident microglia also respond to retinal damage by entering the cell cycle.

**Fig. 6:**
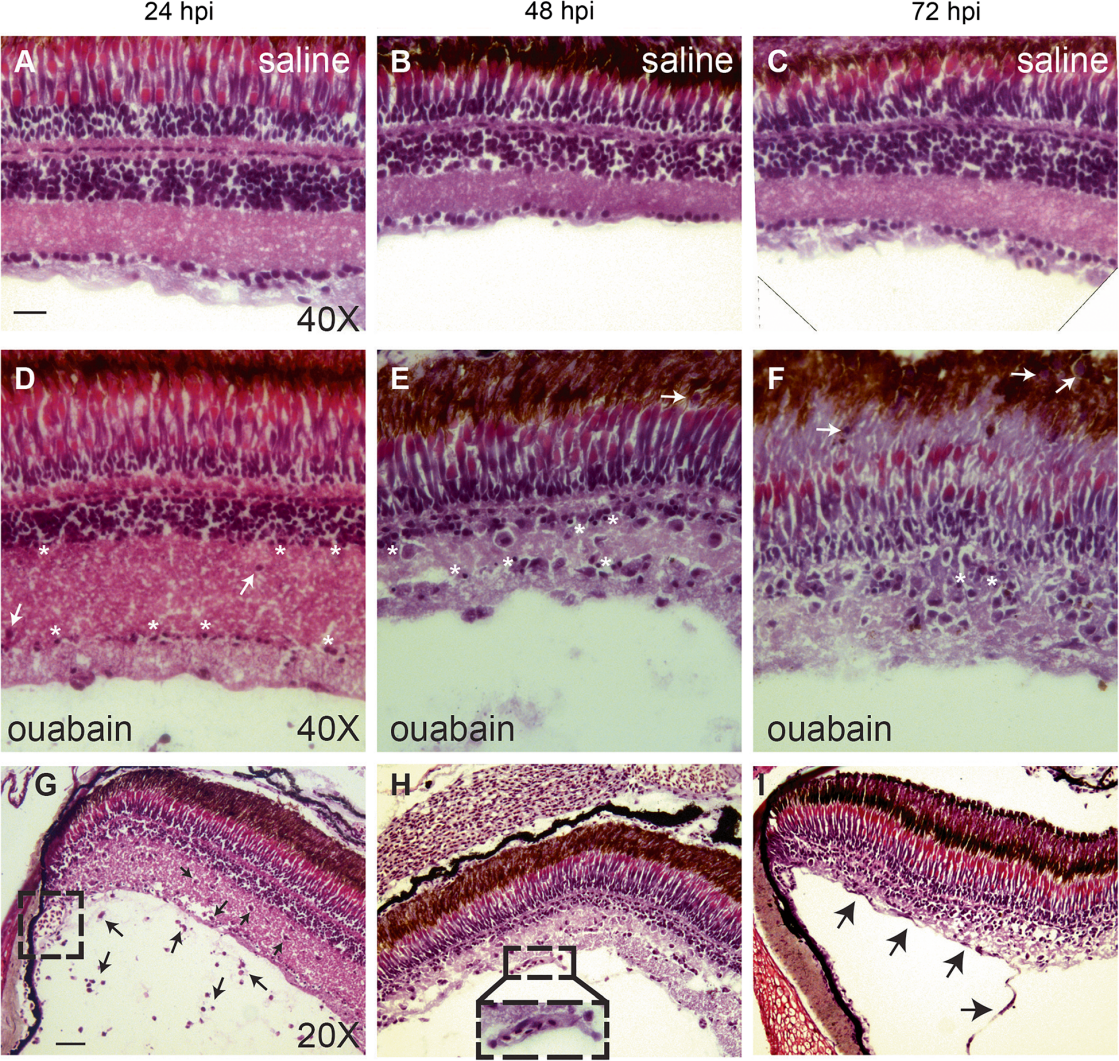
Progression of retinal degeneration and immune cell response visualized by Hematoxylin & Eosin (H&E). H&E staining of cryosections to visualize progression of the ouabain-induced lesion and immune cell accumulation. **A**–**C** Saline-injected controls did not show significant changes in retinal structure; ramified microglia are not readily visualized due to their thin processes with little cytoplasm and integration into retinal layers. **D**–**F** By 24 h post-ouabain injection (24 hpi), the inner retina has begun to swell, pyknotic nuclei are present (asterisks), and ameboid immune cells can be detected within the GCL and INL (**D**, white arrows and **G**, black arrows). Immune cells are visible in the vitreal space at 24 hpi (black arrows, **G**), consistent with invasion of extra-retinal immune cells from the retinal vitreal face. Immune cells accumulate in degenerating retinal tissue, which contains many pyknotic nuclei (asterisks), through 48 and 72 hpi ouabain injection (**E** and **F**). However, immune cells are no longer observed in the vitreal space at 48 and 72 hpi ouabain (**E**, **F**, **H**, **I**). Immune cells present in the degenerating retinal tissue are highly phagocytic, demonstrated by cytoplasmic color, regions of space immediately surrounding them, and the progressive disappearance of extracellular matrix and debris over time (**D**, **E**, **F**). Immune cells appear to invade from structures apical to the retina at 48 and 72 hpi ouabain (white arrows, **E** and **F**). **G**–**I** Evidence of surviving blood vessels at all time points following ouabain injection: at 24 hpi, blood vessel structures at the anterior of the eye adjacent to the peripheral retina are present (dashed box, **G**). Sections of intact retinal blood vessels at the vitreal face of the retina are present at 48 and 72 hpi ouabain injection (dashed box and inset, **H**, and black arrows, **I**). Scale bar in **A** (applies to **A**–**F**) = 20 μm. Scale bar in **G** (applies to **G**–**I**) = 40 μm

To our knowledge, only one study has used a histological method (methylene blue/azure II staining of plastic sections) to document ouabain-induced retinal degeneration in zebrafish [9], and this method does not readily reveal immune cell characteristics. Therefore, to better observe immune cell accumulation concomitant with progression of the ouabain-induced inner retinal lesion and to simultaneously visualize features of responding immune cells, H&E staining of cryosections was used (Fig. 6). Saline-injected controls did not show changes in retinal structure or immune cell accumulation (Fig. 6A–C). However, in damaged retinas, immune cells and the progression of the inner retinal lesion were readily visualized simultaneously using H&E stains (Fig. 6D–I). At 24 hpi, pyknotic nuclei corresponding to dying neurons are visible in the ganglion cell and inner nuclear layer of the retina (Fig. 6D), while immune cells are visible in the vitreal space as well as within the retinal tissue (Fig. 6D, G). By 48 hpi, immune cells are mainly present within regions of degenerating retinal tissue, following the progression of the lesion (Fig. 6E, H). Consistent with this, the presence of extracellular matrix, debris, and pyknotic nuclei decreases from 24 to 72 hpi (Fig. 6D–F). Immune cells continue to accumulate in regions of retinal degeneration from 48 to 72 hpi (Fig. 6E, F), which is likely due to accumulation of dividing immune cells as well as continued immune cell migration from regions apical to the lesion (denoted by white arrows in Fig. 6E, F), indicating that immune cell infiltration into the inner retina continues at least through 72 hpi. Through 72 hpi, evidence of intact vasculature structures at the vitreal face of ouabain injected eyes remains (Fig. 6H, inset, and I, black arrows), suggesting that blood vessels are not immediately and/or fully destroyed by 2 μM ouabain. These vessels could therefore possibly provide infiltrating immune cells access to damaged retinal tissue.

### Identity of S-phase cells at 72 h post-ouabain lesion

**Fig. 7:**
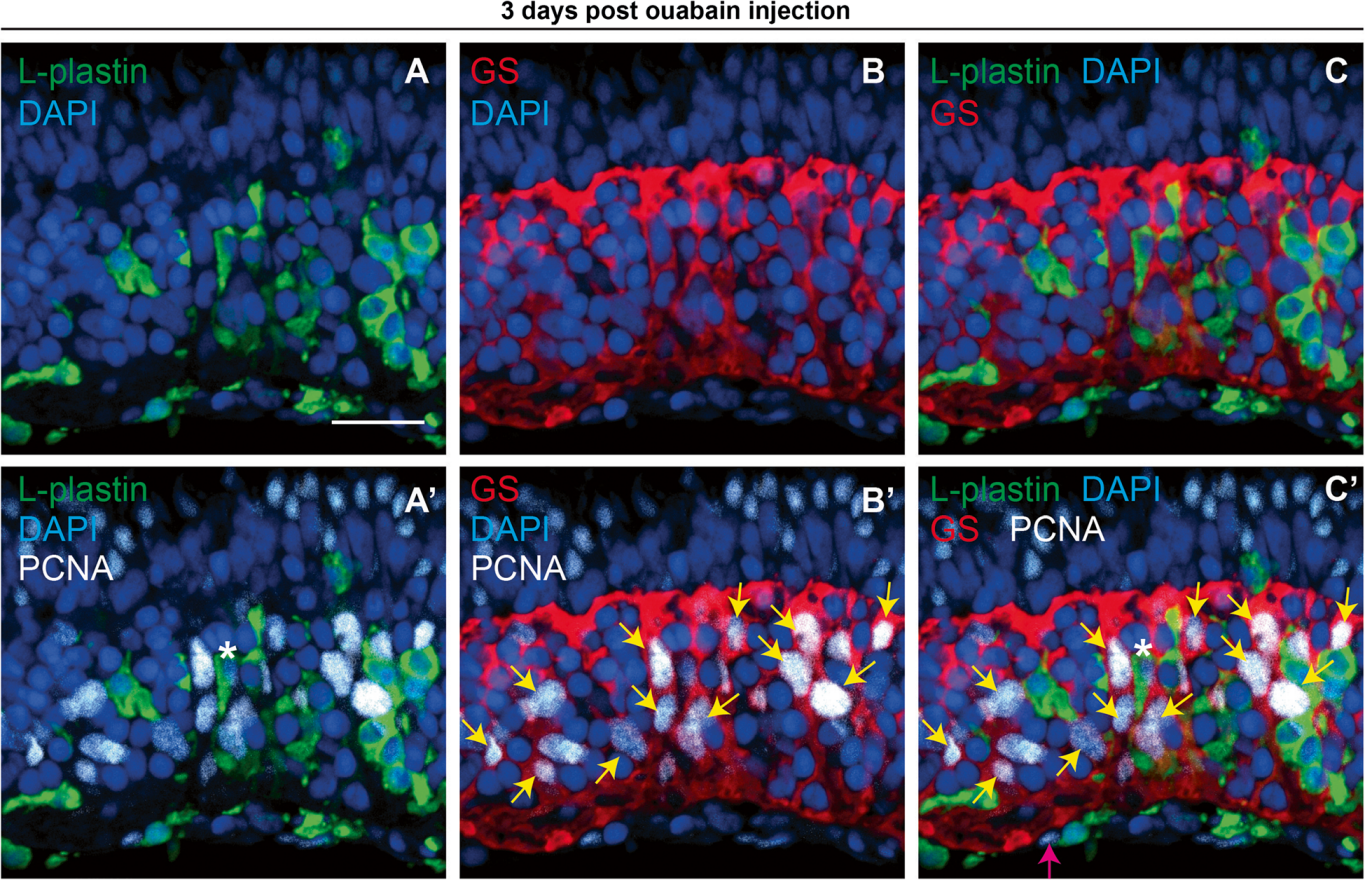
PCNA expression in immune cells and Müller glia at 72 h post-ouabain injection. Representative images of retinal cryosections at 72 h post-ouabain injection (72 hpi) stained for L-plastin to mark immune cells (green), glutamine synthetase to mark Müller glia (GS, red), PCNA (white), and DAPI (blue). **A** L-plastin and DAPI. **B** GS and DAPI. **C** L-plastin, GS, and DAPI. **A’** L-plastin, PCNA, and DAPI. **B’** GS, PCNA, and DAPI. **C’** Four color merge of all stains. Nearly all PCNA+ nuclei can be attributed to immune cells or Müller glia at this time point. Asterisk (*) in **A’** and **C’** denotes L-plastin+, PCNA+ cell. Yellow arrows in **B’** and **C’** are provided to emphasize selected GS+ and PCNA+ cells. Occasionally, we observed PCNA+ nuclei at the vitreal surface at 3 dpi that could not be attributed to L-plastin+ or GS+ cells (**C’**, pink arrow). Scale bar in **A** (applies to all images) = 20 μm

Since Müller glia respond to retinal injury in zebrafish by re-entering the cell cycle [8, 11, 12, 42], and in order to determine the identity of non-leukocyte PCNA+ cells at 72 h post-ouabain injection, we co-stained immune cells in retinal cryosections obtained at this time point with Glutamine Synthetase (GS), which stains Müller glia (Fig. 7). At 72 hpi ouabain injection, nearly all PCNA+ nuclei in central and central-peripheral retina are associated with Glutamine Synthetase+ (GS, Fig. 7B’ and C’, yellow arrows) or L-plastin+ cytoplasm (Fig. 7A’ and C’, asterisk(*)), although we did occasionally detect PCNA+ nuclei in basal retina that were not associated with GS or L-plastin signal (pink arrow, Fig. 7C’). This indicates that by 72 hpi, Mülller glia have re-entered the cell cycle in order to replace neurons lost to the ouabain lesion.

### Identities of immune cells in damaged retinas

**Fig. 8:**
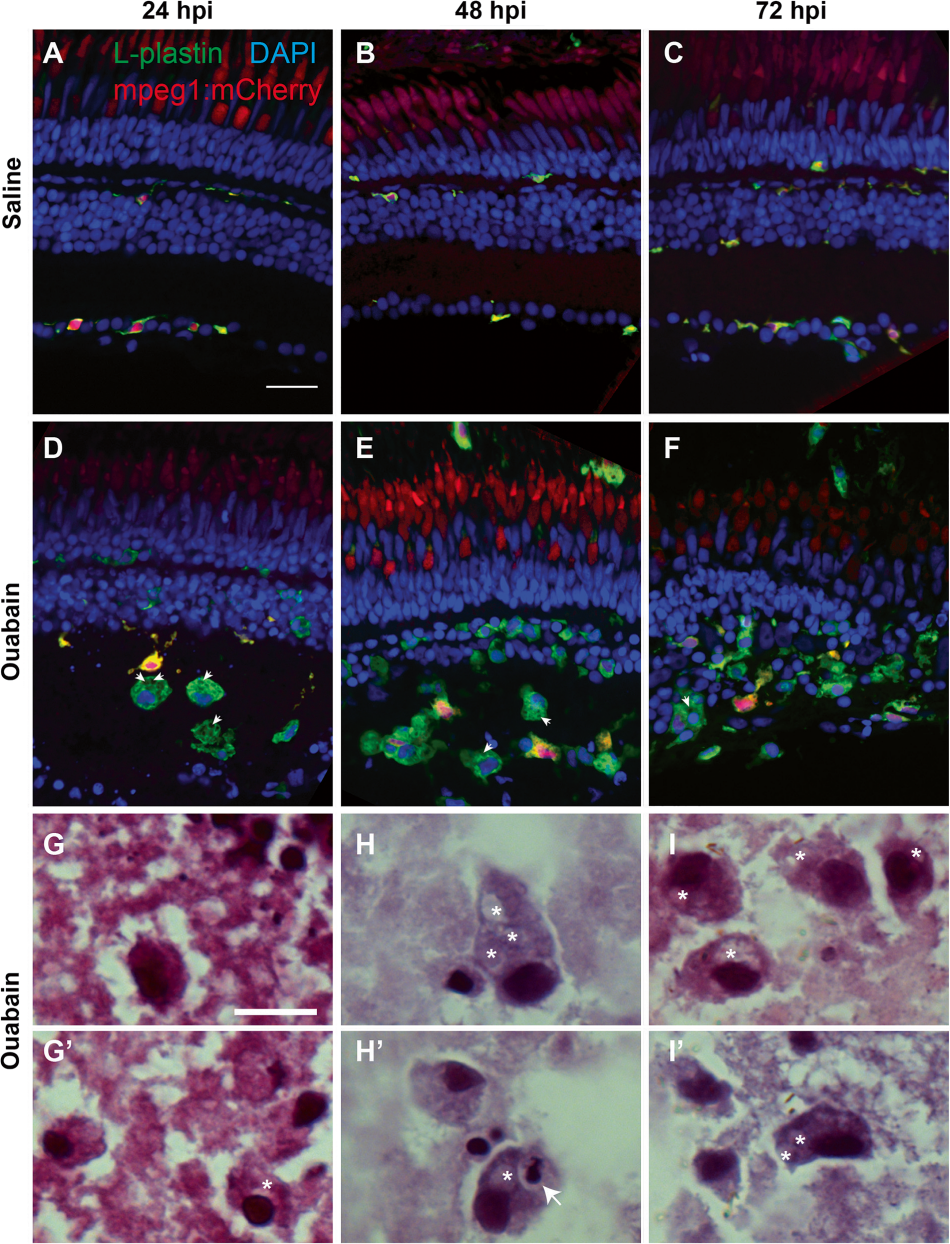
Immune cells responding to ouabain-induced retinal degeneration identify as macrophages. **A**–**F**. Retinal cryosections from transgenic *mpeg1:mCherry* fish following intravitreal injection of saline (**A**–**C**) or ouabain (**D**–**F**) were immunolabeled with L-plastin (green) and counterstained with DAPI (blue). **A**–**C**. In saline-injected controls, all L-plastin+ immune cells (green) co-label with the macrophage marker *mpeg1:mCherry* (red); co-label is yellow (**A**–**C**), indicating ramified microglia. **D**–**F**. Following ouabain injection, ameboid, L-plastin+ immune cells accumulate (green, **D**–**F**), but only a subset of these also express the *mpeg1:mCherry* reporter (yellow, **D**–**F**). Arrowheads indicate vacuoles in selected cells. **G**–**I** High magnification images of retinal cryosections from ouabain damaged retinas stained with H&E reveal classical characteristics of phagocytic macrophages in accumulating immune cells (**G**–**I** and **G’**–**I’**), indicating that these accumulating immune cells are in fact macrophages. These features include irregular shapes of cell bodies and nuclei, cytoplasm similar in color to the environment, space immediately surrounding the cell borders, and the presence of vacuoles (asterisks). **H’** Pyknotic nucleus within the cytoplasm of a macrophage (arrow), indicating phagocytosis of apoptotic cells. The red signal in the ONL in images **A**–**F** is due to autofluorescence from photoreceptors. Scale bar in **A** (applies to **A**–**F**) = 20 μm. Scale bar in **G** (applies to **G**–**I** and **G’**–**I”**) = 10 μm

To determine the identities of the immune cells initially responding to ouabain-induced neuronal cell death, we used cell lineage-specific markers of phagocytic immune cells in cryosections. *Mpeg1* expressing macrophages/microglia are visible at all time points (Fig. 8D–F), but only a fraction of L-plastin+ immune cells present at any of these time points also express the *mpeg1* transgenic reporter (Fig. 8D–F), supporting significant immune cell infiltration to the damaged retina. Despite the absence of *mpeg1* reporter expression, morphological features of the L-plastin+ immune cells in damaged retinal tissue are consistent with a macrophage identity, including irregularly cell-shaped cytoplasm and the presence of numerous vacuoles (Fig. 8D–F, arrowheads). Using H&E staining, we confirmed that these immune cells are macrophages (Fig. 8G–I and G’–I’). Histological features include elongated shape, irregular borders, numerous vacuoles (asterisks, Fig. 8G–I, G’–I’), and granular cytoplasmic inclusions with intensity and color similar to that of extracellular debris, evidence of phagocytosis, and lysosomal degradation (Fig. 8G–I and G’–I’, [43]). H&E staining also revealed numerous vacuoles within macrophages that had phagocytosed pyknotic nuclei (arrow, Fig. 8H’). Very few mpx + neutrophils were detected in cryosections at 24, 48, or 72 h post-ouabain injection (Additional file 1: Figure S5), and H&E staining revealed very few immune cells bearing features of neutrophils (banded or segmented nuclei and pink, granular cytoplasm [43]), indicating that the few neutrophils present in peripheral regions at 12 hpi did not significantly increase in number, and that neutrophils do not comprise a significant portion of responding leukocytes. However, it is possible that the few neutrophils present could represent some of the TUNEL+ immune cells at 24 or 48 hpi (Fig. 2, discussed above), as neutrophils undergo apoptosis shortly after completing their phagocytic functions [43, 44]. We conclude that the immune cells infiltrating the retina in response to ouabain-induced neuron death may be derived from microglia and/or macrophages in nearby tissues and monocytes from circulation that rapidly differentiate to macrophages to perform crucial phagocytic functions. The absence of detectable *mpeg1:mCherry* expression could be explained by delays in expression of the transgenic reporter, or alternatively, that *mpeg1* expression is downregulated in this particular activation state.

### Microglia morphologies and distributions in histologically regenerated retinas

**Fig. 9:**
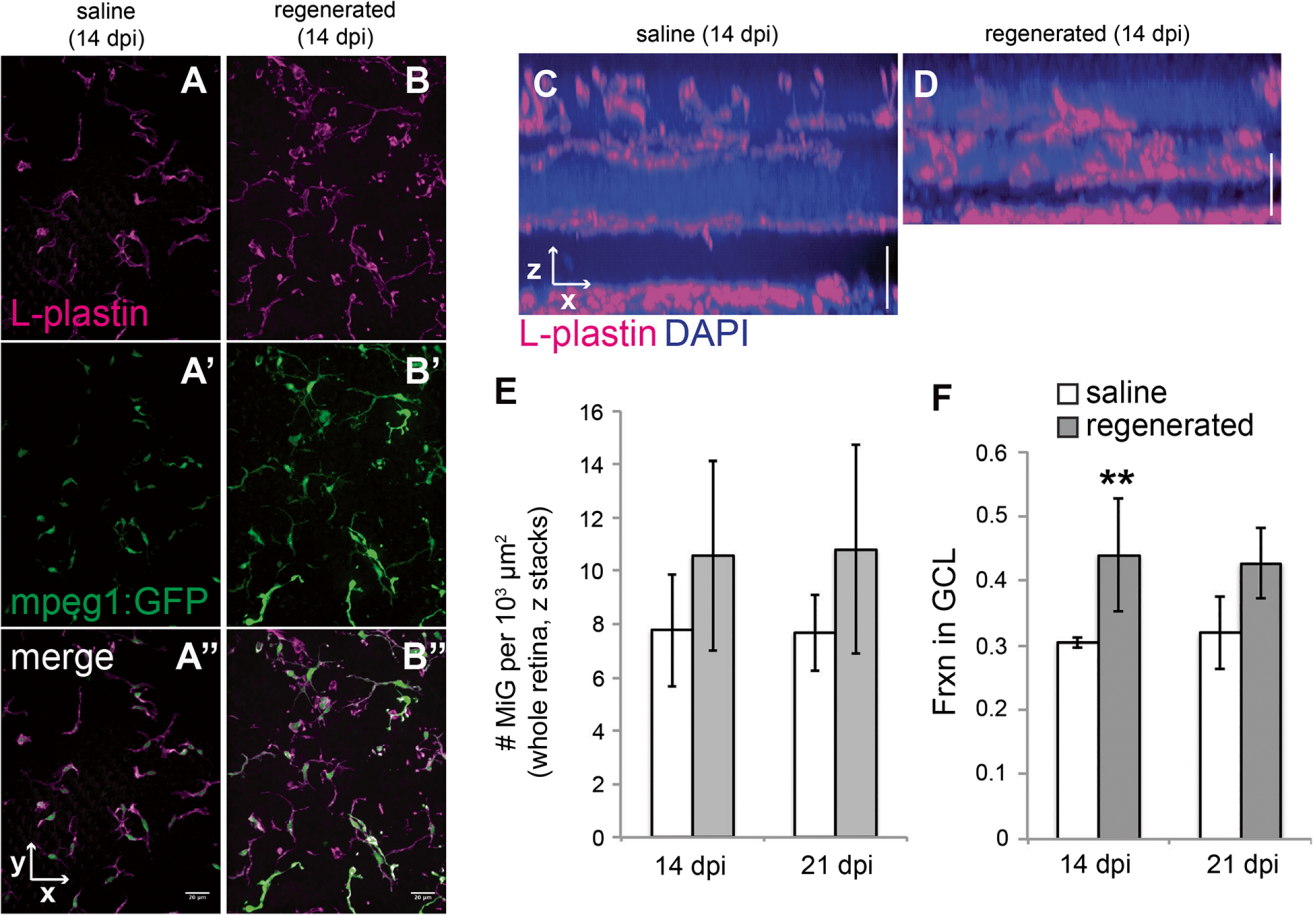
Microglia distribute to regions containing histologically regenerated retinal neurons. **A**–**B**. Images (**A** and **B**) show z projections from saline injected (**A**–**A”**) or regenerated (**B**–**B”**) *mpeg1:GFP* (green) flat-mounted whole retinas labeled with L-plastin (magenta). By 14 dpi, all immune cells in the histologically regenerated retinas express the *mpeg1:GFP* reporter (**B**–**B”**), and the marker L-plastin can be used to label these *mpeg1*:GFP+ immune cells. **C**–**D** Resliced images from whole flat-mounted retinas labeled with L-plastin (magenta) are shown to demonstrate distribution of microglia in control (**C**) and histologically regenerated retinas at 14 dpi ouabain (**D**). **E** Densities of microglia in whole, flat mounted saline injected (*n* = 3) and histologically regenerated retinas (14 (*n* = 4) or 21 dpi (*n* = 3) ouabain). Densities were calculated by counting microglia in individual z stacks obtained from flat mounted retinas, which included retinal layers from the ganglion cell layer to the outer nuclear layer (representing approximately 50–80 μm of depth; Fig. 7A, B in their entirety show representative regions that were quantified). Counts were normalized to 1000 μm² (area) rather than volume because regenerated retinas are thinner than controls. This area represents *x* and *y* directions parallel to the layers of the flattened retina. **F** Fraction of microglia in the ganglion cell layer of saline injected (*n* = 3) and histologically regenerated retinas (14 (*n* = 4) or 21 dpi (*n* = 3) ouabain). **p = 0.048 (two-tailed Student’s *t* test comparing regenerated to saline injected at indicated time point), effect size 1.56. Error bars indicate standard deviation. Scale bar in **A”** (applies to **A**–**A”** and **B**–**B”**) = 20 μm. Vertical scale bar in **C** and **D** = 20 μm

We next investigated microglia morphology and retinal distribution at 14–21 days following ouabain injection. Although there has yet to be a demonstration of functional restoration at these times, we refer to 14 and 21 dpi retinal tissues as “histologically regenerated,” due to the presence of new neurons and the emergence of plexiform layers [9, 10]. These histologically regenerated retinas, however, do show lamination defects. Typical lamination defects are the presence of neuronal nuclei within plexiform layers, particularly the inner plexiform layer; such defects have been referred to as “laminar fusions” [10, 45, 46], some of which resolve over further recovery time [10]. We reasoned that microglia are excellent candidates for involvement in resolution of histological errors and clearing non-functional or apoptotic neurons that may arise during retinal regeneration, and so selected these time points for further analysis. By 14 dpi, all immune cells in the retina express the *mpeg1:GFP* reporter (Fig. 9B–B”). Densities of microglia in histologically regenerated and saline-injected retinas were calculated from whole, flat mounted retinas considering all retinal layers (described in the “Methods” section). Densities of microglia in saline and histologically regenerated retinas were calculated from image stacks obtained from whole, flattened retinas (representative images in Fig. 9A and B). Densities of microglia were slightly elevated in histologically regenerated retinas compared to saline controls, but the differences were not statistically significant (Fig. 9E). However, microglia have not returned to their normal retina distribution patterns and instead remain localized to regions of histologically regenerated retina, particularly the ganglion cell layer (Fig. 9D, F), suggesting that they are indeed performing functional activities in regions containing new neurons.

**Fig. 10:**
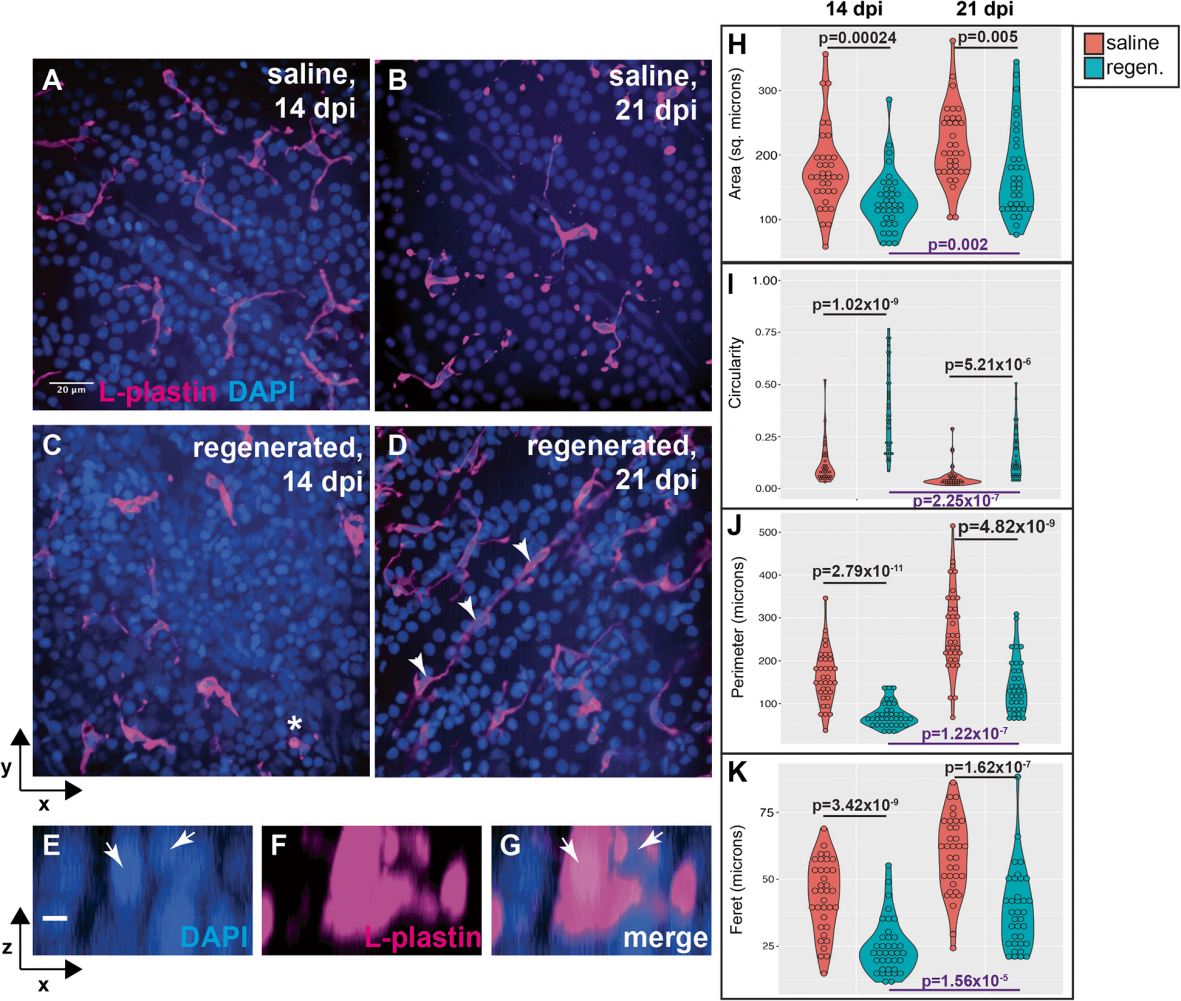
Morphological features of microglia in ganglion cell layer of histologically regenerated retinas. **A**–**D** Images show L-plastin+ microglia (magenta) in the ganglion cell layer (GCL) of saline injected (**A** and **B**) or histologically regenerated retinas at 14 or 21 dpi ouabain (**C** and **D**). Microglia in saline injected retinas appear ramified, displaying long complex processes with multiple tips (**A** and **B**). Microglia in regenerated retinas at 14 dpi appear rounded (ameboid) in shape and have few processes (**C**). In regenerated retinas at 21 dpi, microglia remain more ameboid than those seen in saline injected controls; however, some microglia begin to display more cellular processes (**D**). Microglia in regenerated retinas are occasionally associated with multiple nuclei (asterisk, **C**, resliced projections **E**–**G**). Chains of rod-shaped microglia are observed in regenerated retinas (**D**, arrowheads). **E**–**G** Individual channels in resliced projections of the microglia denoted by a asterisk in (**C**). Arrows indicate DAPI+ nuclei. **H**–**K** Violin plots show distributions of area (**H**), circularity (**I**), perimeter (**J**), and Feret diameter (**K**) of individually traced microglia located in the GCL at 14 and 21 dpi following injection of saline (red plots) or ouabain (regenerated, teal plots). Violin shapes show distribution of all measurements; circles within violin plots represent individual microglia. Area is reported in square microns; circularity as a value from 0 to 1.0 (1.0 indicating perfect circle); perimeter and Feret’s diameter are reported in microns. More information on morphological parameters/measurements is located in the “Methods” section. Indicated *p* values (two-tailed Student’s *t* test) in black compare measurements from regenerated to saline injected at the same time point; *p* values in purple (bottom of graphs) compare measurements from 21 to 14 dpi ouabain. Scale bar in **A** (applies to **A**–**D**) = 20 μm; scale bar in **E** (applies to **E**–**G**) = 5 μm

Further examination of microglia morphologies within the ganglion cell layer at 14 and 21 dpi reveal that microglia retain morphological features of activation rather than ramification (Fig. 10), even though retinal layers have been histologically regenerated by 14 dpi [9, 10, 46]. In control retinas (saline injected), microglia display long, complex processes and appear ramified (Fig. 10A, B), as indicated by long perimeters and increased Feret diameter (Fig. 10J and K). In histologically regenerated retinas, microglia remain ameboid in shape, especially at 14 dpi ouabain (Fig. 10C, D compared to Fig. 10A, B), as indicated by more circular morphology with reduced perimeter and reduced Feret diameter (Fig. 10I, K). Some microglia are associated with multiple nuclei (Fig. 10C asterisk, and E–G), possibly due to phagocytosis of improperly localized, non-functional, or apoptotic neurons. In addition, chains of rod-shaped microglia were observed in regenerated retinas (Fig. 10D). Rod-shaped microglia have been observed following optic nerve transection, which may phagocytose retinal ganglion cell debris and appear to be highly proliferative [47]. In histologically regenerated retinas, between 14 and 21 dpi, distributions of microglia measurements for each parameter begin to shift towards those in control retinas (Fig. 10), indicating dynamic changes are occurring within the microglial population, possibly a trend towards ramification. Collectively, the morphological features of microglia in newly histologically regenerated retinas indicate that rather then returning to a ramified state, microglia remain dynamic as histological features of regenerated retinas continue to improve.

## Discussion

In this study, we examined the immune response to ouabain-induced degeneration of inner retinal neurons in zebrafish, as well as morphology of microglia in histologically regenerated retinas. Key findings include (1) immune cells accumulate rapidly and in large numbers following substantial retinal damage; (2) in addition to local microglia, extra-retinally derived macrophages appear to comprise a significant portion of responding immune cells; and (3) Microglia in histologically regenerated retinas retain morphological features of activation. Collectively, these results indicate that microglia and the immune system are dynamic during retinal degeneration and regeneration and could play important roles in a successful regenerative response in the retina.

### Response of immune cells to damaged retinal tissue

We find that immune cells accumulate rapidly in response to ouabain-induced retinal lesion. We conclude that these immune cells are composed of responding resident microglia as well as extra-retinally derived macrophages, providing a clear identity to the abundant nuclei of unidentified cell type in the inner retina following ouabain damage documented in previous studies [9, 10, 33]. Several publications to date indicate that the innate immune response, particularly macrophages, are crucial to regeneration of a variety of tissues in zebrafish [24–27, 48]. However, it remains unclear to what extent and in which context(s) recruited/peripheral immune cells are involved. In contexts of peripheral nervous system degeneration and regeneration, two studies found a requirement for recruited innate immune cells [24, 25]. In the zebrafish retina, White et al. [26] found that resident microglia were unique in their response to selective rod cell death; recruited phagocytes did not enter the retina. However, these studies were performed in a developmental context (larvae), making it difficult to determine if findings may be tied to a developmental microenvironment or could be shared with degeneration and regeneration in adult animals. Using adult zebrafish following a cell-selective ablation of brain neurons, Oosterhof et al. [49] concluded that peripheral macrophages did not infiltrate the injured brain. This conclusion, however, was based on sequencing results which yielded mainly markers of microglia rather than macrophages (based on mouse orthologues), but only *mpeg1:GFP+* cells were sequenced. Further, the selective neuronal ablation used in the Oosterhof et al. [49] and White et al. [26] studies initiated neuronal cell death through cell-specific drug sensitivity, rather than a more wide-spread and tissue-disrupting chemical insult. In our system, which results in neuron death throughout the entire inner retina of adult fish due to chemical insult, recruited/invading macrophages comprise a significant portion of responding immune cells. Likewise, following cryolesion of the peripheral adult tench retina, infiltrating immune cells were documented [50]. Likely, the executed cell death pathway(s) and resulting molecules released into the tissue environment by dying neurons, as well as the extent of tissue degeneration, are important factors in determining if peripheral immune cells are recruited to the retina.

We also find that a significant portion of immune cells enter the cell cycle upon ouabain-induced retinal injury, similar to findings following acute injury in the zebrafish brain [49] and selective cell death in the retina [26]. PCNA+ immune cells detected at 24 h post-ouabain injection present at locations consistent with ramified retinal microglia (Fig. 4J), as well as those residing at the vitreal face of the ganglion cell layer (Fig. 4D, J), suggest that both infiltrating immune cells and resident microglia enter the cell cycle in response to ouabain-induced retinal lesion. As cell division in responding macrophages weans by approximately 3 days post-injury, PCNA signal is instead detected in non-immune cells, which we have identified to be almost entirely Müller glia (Fig. 7) that are likely entering the cell cycle to replace the damaged neurons. Collectively, this suggests that a relatively large and robust response of phagocytic macrophages and microglia is required to clear dead cell bodies and debris before a regenerative response begins.

### Infiltration of immune cells to degenerating retina suggests systemic inflammatory signals

The act of phagocytosis by macrophages does not necessarily initiate an inflammatory response [51]. However, because our data supports substantial levels of immune cell infiltration into the damaged retina following a ouabain lesion, this indicates that at least some systemic inflammatory signals are received by extra-retinal immune cells, even before the peak of retinal cell death. Acute inflammation appears to be a crucial signal initiating CNS regeneration and specific inflammatory mediators alone may be able to trigger neuronal progenitor proliferation in zebrafish [27]. In our system of retinal damage in zebrafish, as well as others [26, 52], responding microglia/macrophages localize almost exclusively to regions of retinal degeneration, suggesting that inflammatory signals are spatially controlled. In particular, the localization of responding macrophages in our system moves in a wave from the central region originating from the optic nerve head and the vitreal surface of the retina at the periphery, to the inner nuclear layer, which correlates temporally with the progression of the ouabain-induced retinal lesion. Further, the switch in proliferative activity from responding immune cells to Müller glia (discussed above) suggests that inflammation is indeed acute and temporally controlled.

Hallmarks of neurodegenerative disease include chronic inflammation and infiltration of immune cells; however, it is now recognized that these features may have both pathological and beneficial effects [53–56]. In the context of damaged zebrafish retina, however, inflammation appears to be beneficial rather than detrimental, which is in contrast to what has been described in mammals. It is possible that zebrafish have the ability to rapidly switch any harmful inflammatory signals to those that are supportive of regeneration. Alternatively, the molecular aspects of inflammation in zebrafish may differ from that of mammals [57]. Interestingly, aspects of reactive gliosis still exist during retinal regenerative responses in zebrafish [58], suggesting that aspects of harmful inflammation are not completely absent, but supportive signals likely dominate. Consistent with this idea, both pro- and anti-inflammatory cytokines have been found to be important to retinal regeneration in zebrafish following light damage [31, 59] and immune genes were among the top hits yielded by microarray analysis in a context of ongoing rod photoreceptor death and regeneration [60]. Further work is needed to identify key inflammatory signals that are detrimental to regeneration versus those that are supportive. Likely, spatio-temporal control of both pro- and anti-inflammatory mediators is important, and these signals could differ depending on the particular disease or damage system. Importantly, our results support that the immune environment in mammals could potentially be modulated to promote retinal regeneration.

### Morphologically, microglia in histologically regenerated retinas appear functionally active

To date, most studies of inflammation and/or microglia in retinal regeneration have focused on effects on Müller glia proliferation [22, 26]. Further, recent studies that depleted microglia/macrophages prior to or during the initial immune response to neuronal degeneration altered the dynamics and timing of regeneration [24, 26]. However, regeneration of neurons, although delayed, was ultimately recovered. We therefore characterized microglia in histologically regenerated retinas, after the peak of Müller glia proliferation [9], at the time that plexiform layers become evident, but prior to documented restoration of visual function [10]. We found that microglia localize to regions of regenerated neurons, rather than returning to their typical distributions. In addition, microglia retain morphology that indicates activation for several weeks following the initial lesion, suggesting that microglia are active and performing functional roles. It remains to be determined when, or if, microglia return to their original distribution patterns and ramified state, and whether this coincides with restoration of retinal function. Although visually mediated behaviors and ERGs are restored by 60–80 dpi [10, 33], it is not known if ERGs are restored prior to this time point. It has been documented that histological organization of the regenerated retina improves over time [9, 10], and it is possible that this may correlate temporally with recovery of retinal function. Microglia/macrophages are excellent candidates to facilitate improvement of histological organization as they are mobile, highly responsive, and highly phagocytic making them readily able to locate and eliminate apoptotic or non-functional neurons, excess neurons shown to be generated in zebrafish retinal regeneration [10, 61], and/or prune regenerated synaptic processes that arise during retinal regeneration. Therefore, functional roles of microglia/macrophages during retinal regeneration in zebrafish should be explored. These functions may include known roles that are essential to central nervous system development and normal processes [62–69], yet it is also possible that currently unknown or novel functional roles may be involved and are yet to be discovered.

## Conclusions

Interactions of the immune and central nervous system have seen a surge of interest, supporting the idea of immune system influence on regenerative potential [70]. With goals to regenerate damaged retinal tissue in humans, further work in investigating neuro-immune interactions during successful retinal regeneration, which occurs in a variety of vertebrate organisms including teleost fish, is crucial. The present work provides a framework for future studies and confirms that the zebrafish retina is an excellent model to study immune-neuron interactions during neuronal regeneration. This will allow us to uncover key inflammatory signals and the function of the innate immune system in the initiation and successful execution of retinal regeneration. Further, this will allow us to probe the functional roles of microglia in the retina, both in normal and regenerative contexts.

## Additional file

Additional file 1: **Figure S1**. A–A”. To assess co-label of *mpeg1:GFP* and *mpeg1:mCherry* transgenes in retina, double transgenic embryos from a cross of gl22 Tg x gl23 Tg fish at 3 dpf were fixed, washed, and eyes removed then mounted for imaging. A z series (5 μm step size) was obtained. Images show selected z-projections from whole eyes; white lines indicate the eye boundary. A. mpeg1:GFP signal. A’ mpeg1:mCherry signal. A” Merge to show colabel. Scale bar in A” = 20 μm. Whole retinas from adult mpeg1:GFP and mpeg1:mCherry fish were stained for L-plastin. Images show expression of individual transgenes with L-plastin (magenta, B’, and C’). Essentially all transgene signal coincides with L-plastin (B” and C”). Scale bars in B” and C= 100 μm. **Figure S2.** Retinal cryosections corresponding to peripheral (A) or central regions (B), adjacent to the optic nerve head (onh, denoted by **) at 12 h post-injection (12 hpi) saline. Cryosections were stained for PCNA (green), L-plastin (magenta), and DAPI (blue). Scale bar in B = 20 μm, applies to both images. **Figure S3.** Retinal cryosections corresponding to peripheral (A) or central regions (B, adjacent to onh, denoted by **) at 12 hpi ouabain from mpeg1:mCherry transgenic fish stained for mpx (green) and DAPI (blue). Scale bar in B = 20 μm, applies to both images. **Figure S4.** Images show retinal cryosections from mpeg1:mCherry fish following intravitreal saline (A) or ouabain (B) injection at 72 hpi. Cryosections were labeled with anti-phosphorylatedhistone 3 (PH3, green) and DAPI (blue). Arrows indicate PH3+ nuclei. Red signal in the outer retina is autofluorescence from photoreceptors. Scale bar in A = 20 μm, applies to A and B. **Figure S5.** Images show retinal cryosections following intravitreal injection of ouabain at 24, 48, and 72 hpi. Cryosections were stained for mpx (red) and DAPI (blue). Scale bar = 20 μm, applies to all images. (PDF 1222 kb)

## Abbreviations

CMZ: Ciliary marginal zone
DAPI: 4′,6-diamidino-2-phenylindole
dpi: Days post-injection
GCL: Ganglion cell layer
H&E: Hematoxylin and Eosin
hpi: Hours post-injection
INL: Inner nuclear layer
MG: Müller glia
ONL: Outer nuclear layer
PCNA: Proliferating cell nuclear antigen
PKC: Protein kinase c
TUNEL: Terminal deoxynucleotidyl transferase dUTP nick end labeling
