# Supplementary material for: Dynamic changes in microglial and macrophage characteristics during degeneration and regeneration of the zebrafish retina
Source: J Neuroinflammation. 2018 May 28;15:163. doi: 10.1186/s12974-018-1185-6 (PMC5971432; doi:10.1186/s12974-018-1185-6)
Supplement: Supplementary file 1 — Figure S1. A–A”. To assess co-label of mpeg1:GFP and mpeg1:mCherry transgenes in retina, double transgenic embryos from a cross of gl22 Tg x gl23 Tg fish at 3 dpf were fixed, washed, and eyes removed then mounted for imaging. A z series (5 μm step size) was obtained. Images show selected z-projections from whole eyes; white lines indicate the eye boundary. A. mpeg1:GFP signal. A’ mpeg1:mCherry signal. A” Merge to show colabel. Scale bar in A” = 20 μm. Whole retinas from adult mpeg1:GFP and mpeg1:mCherry fish were stained for L-plastin. Images show expression of individual transgenes with L-plastin (magenta, B’, and C’). Essentially all transgene signal coincides with L-plastin (B” and C”). Scale bars in B” and C= 100 μm. Figure S2. Retinal cryosections corresponding to peripheral (A) or central regions (B), adjacent to the optic nerve head (onh, denoted by **) at 12 h post-injection (12 hpi) saline. Cryosections were stained for PCNA (green), L-plastin (magenta), and DAPI (blue). Scale bar in B = 20 μm, applies to both images. Figure S3. Retinal cryosections corresponding to peripheral (A) or central regions (B, adjacent to onh, denoted by **) at 12 hpi ouabain from mpeg1:mCherry transgenic fish stained for mpx (green) and DAPI (blue). Scale bar in B = 20 μm, applies to both images. Figure S4. Images show retinal cryosections from mpeg1:mCherry fish following intravitreal saline (A) or ouabain (B) injection at 72 hpi. Cryosections were labeled with anti-phosphorylatedhistone 3 (PH3, green) and DAPI (blue). Arrows indicate PH3+ nuclei. Red signal in the outer retina is autofluorescence from photoreceptors. Scale bar in A = 20 μm, applies to A and B. Figure S5. Images show retinal cryosections following intravitreal injection of ouabain at 24, 48, and 72 hpi. Cryosections were stained for mpx (red) and DAPI (blue). Scale bar = 20 μm, applies to all images. (PDF 1222 kb) [file 12974_2018_1185_MOESM1_ESM.pdf]

Supporting Information

Supplementary Figure 1

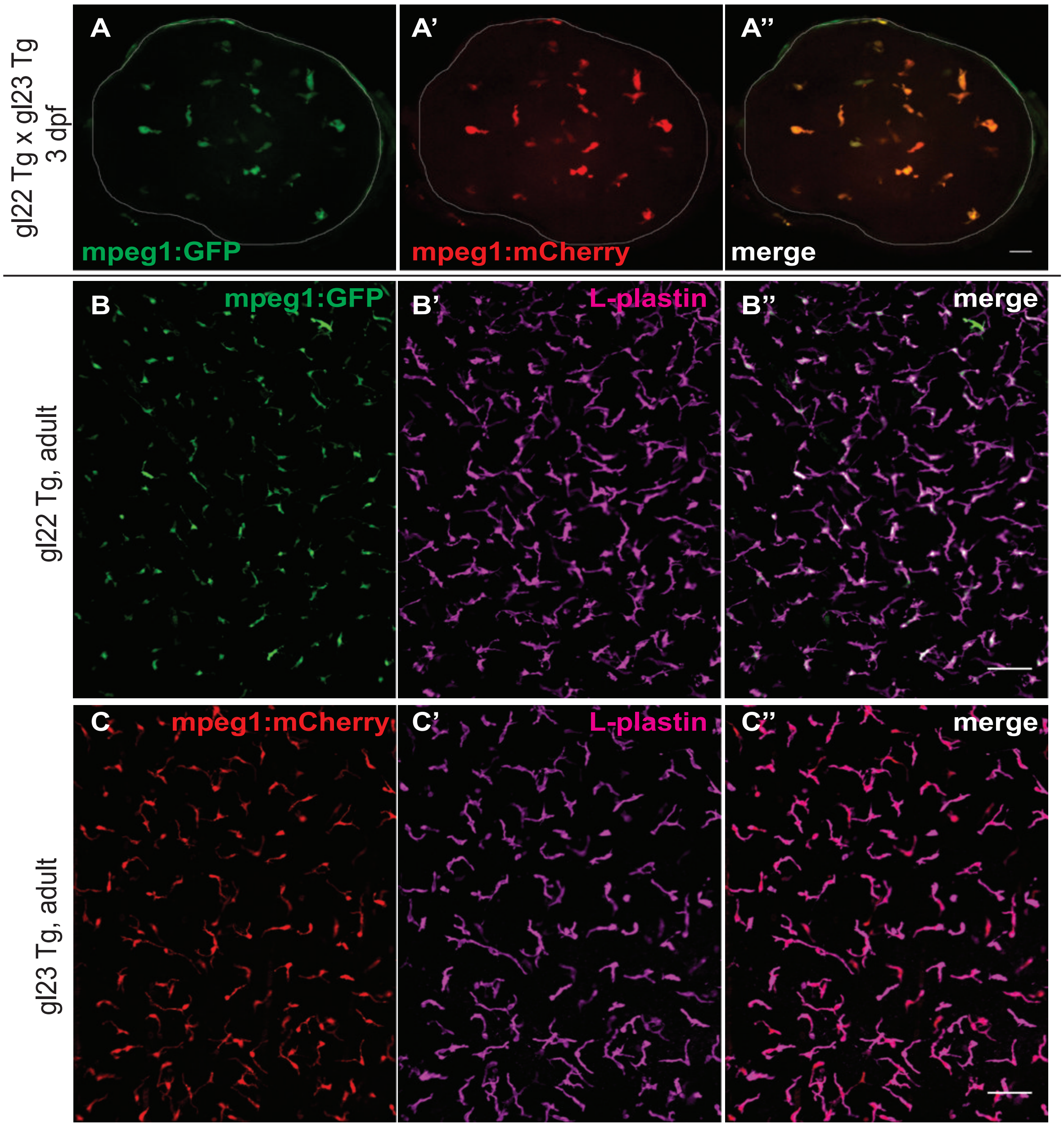

A-A''. To assess co-label of *mpeg1:GFP* and *mpeg1:mCherry* transgenes in retinal tissue, embryos were obtained from a cross of gl22 Tg x gl23 Tg fish. At 3 days post-fertilization (3 dpf), embryos were screened for expression of both *mpeg1:GFP* and *mpeg1:mCherry* transgenes. Embryos were fixed, washed, and eyes removed then mounted for imaging. A z series (5 micron step size) was obtained. Images show selected z-projections from whole eyes, with white lines indicating the eye boundary. A. mpeg1:GFP signal. A'. mpeg1:mCherry signal. A''. Merge to show colabel. Scale bar in A'' = 20 microns. Whole retinas from adult mpeg1:GFP (gl22 Tg) and mpeg1:mCherry (gl23 Tg) were stained for L-plastin. Images show expression of of each individual transgene (B, mpeg1:GFP, green or C, mpeg1:mCherry, red) with L-plastin stain (magenta, B' and C'). In both transgenic lines, essentially all transgene signal coincides with L-plastin (B'' and C''). Scale bars in B'' and C'' = 100 microns.

Supplementary Figure 2

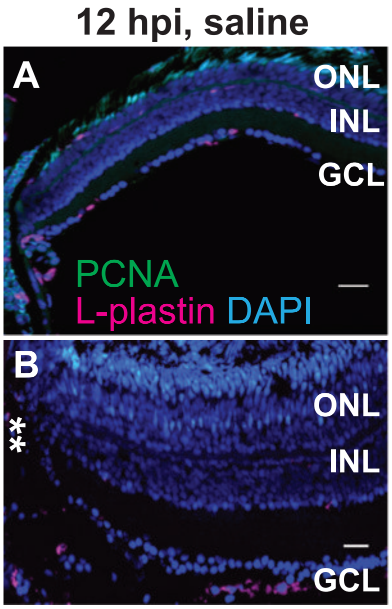

Images show retinal cryosections corresponding to peripheral (A) or central regions (B), adjacent to the optic nerve head (denoted by **) at 12 hours post injection (12 hpi) of saline. Cryosections were stained for PCNA (green), L-plastin (magenta), and DAPI (blue). Saline injection did not induce substantial PCNA expression at 12 hpi. Scale bar in B = 20 microns and applies to both images.

Supplementary Figure 3

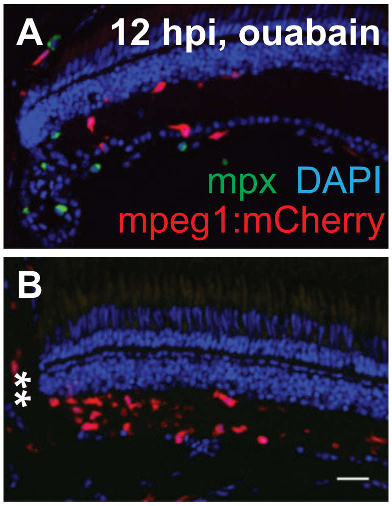

Images show retinal cryosections corresponding to peripheral (A) or central regions (B, adjacent to the optic nerve head, denoted by **) at 12 hours post injection (12 hpi) of ouabain. Cryosections from *mpeg1:mCherry* transgenic fish were stained for mpx (green) and DAPI (blue). Few neutrophils can be seen in peripheral regions. Scale bar in B = 20 microns and applies to both images.

Supplementary Figure 4

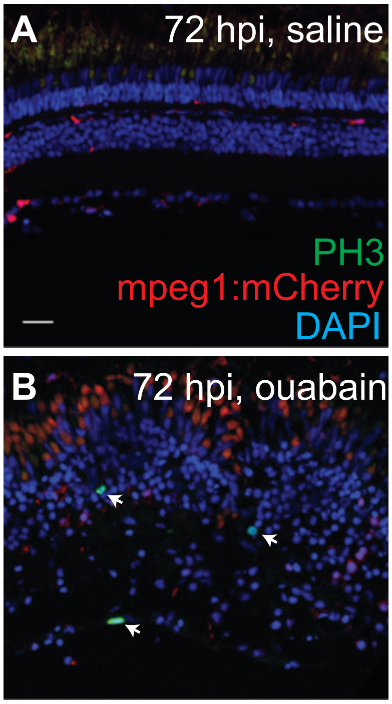

Images show retinal cryosections from *mpeg1:mCherry* fish following intravitreal saline (A) or ouabain (B) injection at 72 hours post injection (hpi). Cryosections were labeled with anti-phosphorylated histone 3 (PH3, green) and DAPI (blue). Arrows indicate PH3+ nuclei. Red signal in the outer retina is autofluorescence from photoreceptors. Scale bar in A = 20 microns and applies to A and B.

Supplementary Figure 5

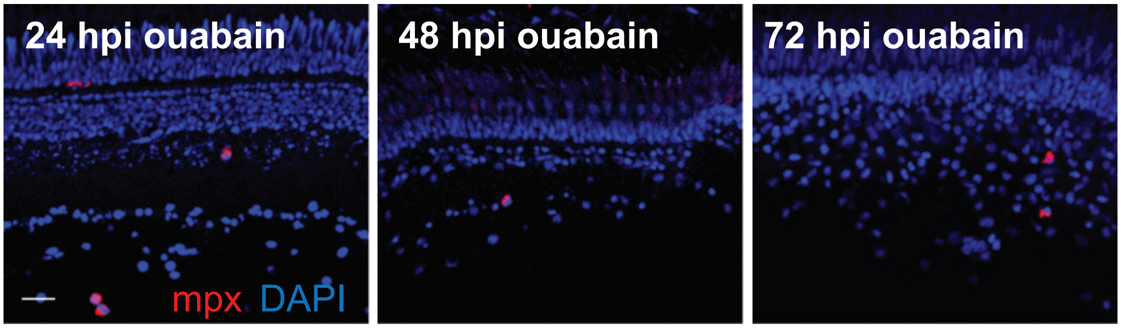

Images show retinal cryosections from zebrafish following intravitreal injection of ouabain, at 24, 48, and 72 hours post injection (hpi). Cryosections were labeled with the neutrophil specific mpx antibody (red) and DAPI (blue). Scale bar = 20 microns and applies to all images.
